# Economical production of *Pichia pastoris* single cell protein from methanol at industrial pilot scale

**DOI:** 10.1186/s12934-023-02198-9

**Published:** 2023-09-28

**Authors:** Jiao Meng, Shufan Liu, Le Gao, Kai Hong, Shuguang Liu, Xin Wu

**Affiliations:** 1 Tianjin Institute of Industrial Biotechnology, Chinese Academy of Sciences, National Technology Innovation Center of Synthetic Biology, No. 32, Xiqi Road, Tianjin Airport Economic Park, 300308 Tianjin, Tianjin, China; 2 Ningxia Future Biotechnology Co., Ltd, Jingsan Road, Ningdong Linhe Industrial Zone, Ningdong Town, Ningxia, China

**Keywords:** *Pichia pastoris*, Methanol, Single cell protein, Engineering, Economical benefit

## Abstract

**Background:**

Methanol, synthesized from CO_2_, is a potentially sustainable one-carbon (C1) resource for biomanufacturing. The use of methanol as a feedstock to produce single cell protein (SCP) has been investigated for decades as an alternative to alleviate the high global demand for animal-derived proteins. The methylotrophic yeast *Pichia pastoris* is an ideal host for methanol-based SCP synthesis due to its natural methanol assimilation ability. However, improving methanol utilization, tolerance to higher temperature, and the protein content of *P. pastoris* are also current challenges, which are of great significance to the economical industrial application using methanol as a feedstock for SCP production.

**Results:**

In the present work, adaptive laboratory evolution (ALE) has been employed to overcome the low methanol utilization efficiency and intolerance to a higher temperature of 33 °C in *P. pastoris*, associated with reduced carbon loss due to the lessened detoxification of intracellular formaldehyde through the dissimilation pathway and cell wall rearrangement to temperature stress resistance following long-term evolution as revealed by transcriptomic and phenotypic analysis. By strengthening nitrogen metabolism and impairing cell wall synthesis, metabolic engineering further increased protein content. Finally, the engineered strain via multi-strategy produced high levels of SCP from methanol in a pilot-scale fed-batch culture at 33 °C with a biomass of 63.37 g DCW/L, methanol conversion rate of 0.43 g DCW/g, and protein content of 0.506 g/g DCW. SCP obtained from *P. pastoris* contains a higher percentage of protein compared to conventional foods like soy, fish, meat, whole milk, and is a source of essential amino acids, including methionine, lysine, and branched-chain amino acids (BCAAs: valine, isoleucine, leucine).

**Conclusions:**

This study clarified the unique mechanism of *P. pastoris* for efficient methanol utilization, higher temperature resistance, and high protein synthesis, providing a *P. pastoris* cell factory for SCP production with environmental, economic, and nutritional benefits.

**Supplementary Information:**

The online version contains supplementary material available at 10.1186/s12934-023-02198-9.

## Background

To meet the global demand for animal-derived proteins, the world will need to produce 1,250 million tons of meat and dairy products annually. However, due to the low efficiency in converting feed to meat and dairy products, the growing human demand for proteins will not be met sustainably by increasing meat and dairy production [1, 2]. Although artificial meat and plant-based meat substitutes are currently developing rapidly, higher production costs and poorer taste make them less competitive than animal-based proteins produced through traditional livestock farming [3, 4]. Therefore, it is still necessary to find and develop new protein resources. Single cell protein (SCP), which is produced in algae (protein content is approximately 60–70%), fungi (protein content is approximately 30–50%) and bacteria (protein content is approximately 50–80%) cells, is a potential option [5–7]. The unique substrate (such as CO_2_ or methane) utilization ability and high growth rate of these microorganisms make the production process of SCP more efficient and sustainable than traditional agriculture [8].

**Fig. 1:**
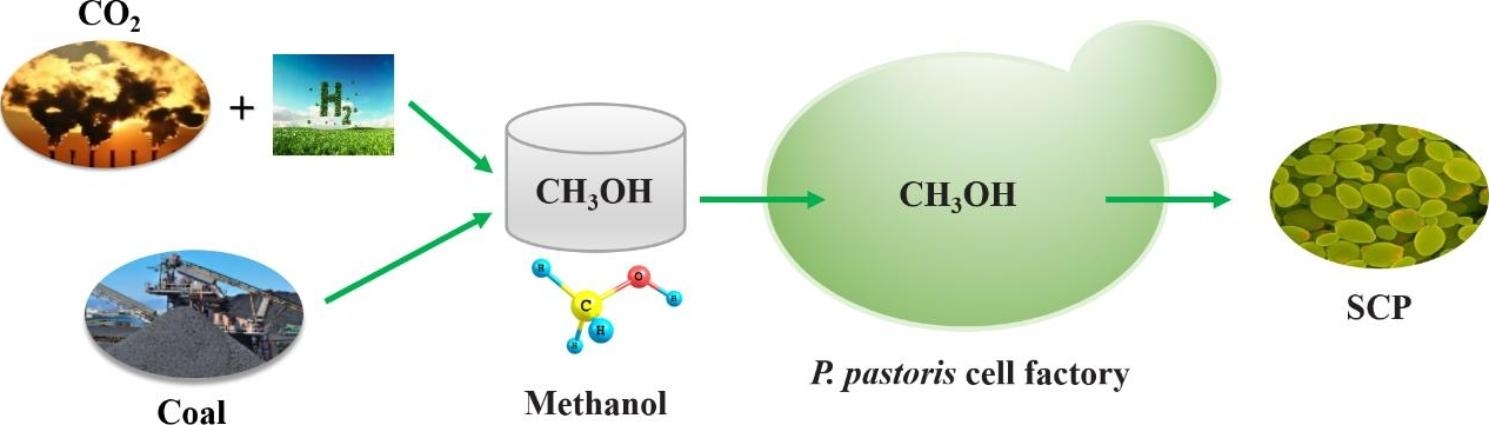
The concept of methanol-based SCP production in *P. pastoris*. Methanol can be produced from low-quality coal and CO_2_ hydrogenation in large quantities, representing a sustainable production mode using methanol as a feedstock for future biomanufacturing and one of the potential ways to achieve global carbon neutrality

The concentration of CO_2_ in the global atmosphere is increasing at an alarming rate, and the climate and greenhouse effect have affected human survival and development. The growing environmental crisis has exacerbated the need for a shift away from the traditional petrochemical economy towards sustainable feedstocks. One-carbon (C1) compound assimilation by microorganisms has emerged as a promising approach in abating climate change [9–11]. Methanol is regarded as an ideal C1 resource for chemical industry and biomanufacturing, since it can be produced not only from low-quality coal, but also from CO_2_ and hydrogen (electrolyzed water) in large quantities by electrocatalysis or photocatalysis, which is considered as one of the most potential ways to achieve global carbon neutrality. As a liquid, methanol is easier to transport and store compared to those gaseous C1 compounds [12, 13]. Furthermore, methanol has a high degree of reduction that can provide more driving force for product biosynthesis [14]. Therefore, the cheap and abundant sources, flexible production processes, compatibility with current transportation and fermentation infrastructure, and high reduction potential make methanol an attractive substrate for SCP biosynthesis (Fig. 1).

In recent years, researchers have tried to construct a methanol assimilation pathway by constructing heterologous and artificial utilization pathways, and achieved the conversion of methanol to central metabolism in model microorganisms such as *Escherichia coli*, *Saccharomyces cerevisiae* and *Corynebacterium glutamicum* [15–17]. However, these engineered strains still cannot grow use methanol as sole carbon source. Recently, Chen et al. realized the growth of *E. coli* using methanol as the sole carbon through the combination of metabolic engineering and adaptive laboratory evolution (ALE). However, the doubling time of this strain is up to 8.5 h, and the maximum optical density (OD_600_) is only about 2, which greatly limits the methanol metabolism rate and product synthesis efficiency [11]. Notably, native methylotrophic yeast, *Pichia pastoris* (also known as *Komagataella phaffii*), might be an ideal host for methanol biotransformation given its natural capacity in methanol assimilation system. Particularly, *P. pastoris* is an important industrial methanol yeast with GRAS (Generally Recognized as Safe) certification by the U.S. Food and Drug Administration (FDA), which is widely used in the production of recombinant proteins [18, 19]. With the whole genome sequencing and subsequent annotation of commonly used strains CBS7435 and GS115, as well as the development of more accurate genome editing system, the research on obtaining target products through engineering *P. pastoris* based on methanol has become a hot spot [20–22]. So far, some chemicals, such as lactic acid, non-animal chondroitin sulfate, malic acid and free fatty acids etc., have been synthesized from methanol in *P. pastoris* after metabolic rewiring [23–26]. However, the complex methanol metabolism pathway and the toxicity of intracellular formaldehyde led to the low methanol utilization efficiency in natural *P. pastoris*, which is not conducive to the synthesis of target products [27, 28].

Since decades, *P. pastoris* has been using methanol as a feedstock for producing SCP [29], but improving the conversion of methanol into biomass has always been problematic. Further, since the fermentation temperature of *P. pastoris* is actually higher than its growth temperature (28–30 °C), intolerance to higher temperature usually reduces substrate conversion efficiency and increases cooling costs in the industrial production process [30]. Thus, using a higher-temperature-resistant *P. pastoris* for SCP biosynthesis is a potential way to reduce production costs. Importantly, the nutritional value of SCP is closely related to the protein content of *P. pastoris*, a *P. pastoris* strain with high-protein content will be more competitive with other protein sources [1, 2]. As a result, we can conclude that one of the key considerations of economic production of SCP based on methanol is to develop a *P. pastoris* cell factory capable of utilizing methanol efficiently, tolerating higher temperature, and producing high amounts of protein.

In the current study, ALE was used to obtain a *P. pastoris* strain with high methanol utilization efficiency and tolerance to a higher temperature of 33 °C (this temperature was selected in consideration of both biomass and protein content). Transcriptome and intracellular formaldehyde analyses showed that less formaldehyde was detoxified through the dissimilation pathway, ultimately resulting in a reduction in carbon loss in the evolved strain. It was also found that effective induction of cell wall remodeling was necessary to protect *P. pastoris* cells from temperature stress during evolution. Following that, metabolic engineering was used to improve protein content in this evolved host by improving nitrogen metabolism and impairing cell wall synthesis, with the best strain achieving high-level production of SCP from sole methanol in a pilot-scale fed-batch culture at 33 °C. In addition to high protein content, SCP from *P. pastoris* is also a source of essential amino acids. This study successfully elucidated the unique mechanism of methanol utilization efficiently, higher temperature tolerance and high protein synthesis in *P. pastoris*, providing a *P. pastoris* cell factory for economical industrial application using methanol as a substrate for SCP production.

## Materials and methods

### Strains, media and cultivation

All strains and plasmids used in this study are listed in Table 1. The plasmid pPICZ-Cas9-gGUT1 [22] was kindly offered by Prof. Yongjin Zhou from Dalian Institute of Chemical Physics (DICP), Chinese Academy of Sciences, and other *P. pastoris* strains and plasmids were stored in our lab or constructed in this study. *E. coli* DH5α used as the host for plasmid construction were cultivated at 37 °C in a rotatory shaker at 220 rpm in Luria-Bertani (LB) consisting of 5 g/L yeast extract, 10 g/L tryptone, and 10 g/L NaCl with 50 µg/mL zeocin to maintain plasmids. Unless otherwise specified, *P. pastoris* strains were cultivated in YPD medium at 30 or 33 °C in a rotatory shaker at 220 rpm consisting of 20 g/L glucose, 20 g/L peptone, and 10 g/L yeast extract. And 100 µg/mL zeocin was added to YPD medium for screening transformants. The composition of the Delft basic salt medium used for cell cultivation with methanol as single carbon source were 14.4 g/L KH_2_PO_4_, 7.5 g/L (NH_4_)_2_SO_4_, 0.5 g/L MgSO_4_•7H_2_O, 1 ml/L vitamin solution, and 2 ml/L trace metal solution [22, 31].

**Table 1:** Strains and plasmids used in this study

Strains and Plasmids	Relevant Characteristics	Sources
***P. pastoris***		
X-33	Wild type, Mut^+^	Lab stock
HTX-33	Derived from X-33 through ALE	This study
HTX-33-GDH1	Derived from HTX-33, PNSII-3::P_AOX1_-GDH1-T_ADH2_	This study
HTX-33-GLN1	Derived from HTX-33, PNSI-2::P_DAS2_-GLN1-T_ADH2_	This study
HTX-33-GDH1-GLN1	Derived from HTX-33, PNSII-3::P_AOX1_-GDH1-T_ADH2_; PNSI-2::P_DAS2_-GLN1-T_ADH2_	This study
HTX-33-Δ*PAS_chr4_0305*	Derived from HTX-33, Δ*PAS_chr4_0305*	This study
HTX-33-GLN1-Δ*PAS_chr4_0305*	Derived from HTX-33, PNSI-2:: P_DAS2_-GLN1-T_ADH2_; Δ*PAS_chr4_0305*	This study
***E. coli***		
DH5a	F^−^, φ80*lacZ* Δ*M15*, Δ(*lacZYA-argF*)*U169*, *deoR*, *recA1*, *endA1*, *hsdR17*(rk^−^, mk^+^), *phoA*, *supE44*, *λ*^−^, *thi-1*, *gyrA96*, *relA1*	Lab stock
**Plasmids**		
pPICZ-Cas9-gGUT1	ori, Amp, Zeocin, T_DAS1_-Cas9-P_HTX1_-GUT1-gRNA2-T_AOX_	(22)
pPICZ-Cas9-gPNSI-2	ori, Amp, Zeocin, T_DAS1_-Cas9-P_HTX1_- PNSI-2-gRNA-T_AOX_	This study
pPICZ-Cas9-gPNSII-3	ori, Amp, Zeocin, T_DAS1_-Cas9-P_HTX1_- PNSII-3-gRNA-T_AOX_	This study
pPICZ-Cas9-g*PAS_chr4_0305*	ori, Amp, Zeocin, T_DAS1_-Cas9-P_HTX1_-*PAS_chr4_0305*-gRNA-T_AOX_	This study

### Plasmids and strains construction

The method used for genetic manipulation in the chromosome of *P. pastoris* was based on the utilization of CRISPR-Cas9 meditated genome editing system as described previously [22]. To construct gRNA expression plasmids, 20 bp target sequences of gRNAs for genome targeting were designed using a user-friendly online web tool (CRISPR RGEN Tools, https://www.rgenome.net/). As for construction of plasmid pPICZ-Cas9-gPNSII-3, the Cas part and gRNA part were obtained by PCR amplification with primers *gPNSII-3-1 F/gPNSII-3-1R* and *gPNSII-3-2 F/gPNSII-3-2R* using the plasmid pPICZ-Cas9-gGUT1(containing Cas9 and GUT1-gRNA under the control of bidirectional promoter P_HTX1_) as a template. The two parts were fused by Gibson assembly to yield plasmid pPICZ-Cas9-gPNSII-3. Transformations were screened on LB agar plate with 50 µg/mL Zeocin. The plasmids pPICZ-Cas9-gPNSI-2 and pPICZ-Cas9-g*PAS_chr4_0305* were constructed based on the same method using primers *gPNSI-2-1 F/gPNSI-2-1R*, *gPNSI-2-2 F/gPNSI-2-2R*, *gPAS_chr4_0305-1 F/gPAS_chr4_0305-1R* and *gPAS_chr4_0305-2 F/gPAS_chr4_0305-2R*, respectively.

To construct GDH1-donor DNA, the upstream part, promoter part, GDH1 part, terminator part and downstream part, were amplified from the *P. pastoris* genome using primers *PNSII-3-up-F/PNSII-3-up-R*, *PAOX1-F/PAOX1-R*, *GDH1-F/GDH1-R*, *TADH2 (GDH1)-F/TADH2 (GDH1)-R* and *PNSII-3-down-F/PNSII-3-down-R*, respectively. These five fragments were then fused by fusion PCR to obtain GDH1-donor DNA cassettes. As for GLN1-donor DNA, the upstream part, promoter part, GLN1 part, terminator part and downstream part, were amplified from the *P. pastoris* genome using primers *PNSI-2-up-F/PNSI-2-up-R*, *PDAS2-F/PDAS2-R*, *GLN1-F/GLN1-R*, *TADH2 (GLN1)-F/TADH2 (GLN1)-R* and *PNSI-2-down-F/PNSI-2-down-R*, respectively. The five fragments were then fused by fusion PCR to yield GLN1-donor DNA cassettes. To construct *PAS_chr4_0305*-donor DNA, fragments upstream and downstream of the *PAS_chr4_0305* gene were amplified from the *P. pastoris* genome using primers *PAS_chr4_0305-up-F*/*PAS_chr4_0305-up-R* and *PAS_chr4_0305-down-F*/*PAS_chr4_0305-down-R*, respectively. The upstream and downstream fragments were then fused and amplified by fusion PCR to create *PAS_chr4_0305*-donor cassettes.

Finally, the gRNA expression plasmid and the corresponding donor DNA were co-transformed into the competent cells of *P. pastoris*, respectively, and the transformed cells were grown for three days on YPD plates containing 100 µg/mL Zeocin. The mutants were verified by PCR and further confirmed by gene sequencing. All primers used for strains and plasmids construction are listed in Supplementary Table 1.

### Adaptive laboratory evolution

**Fig. 2:**
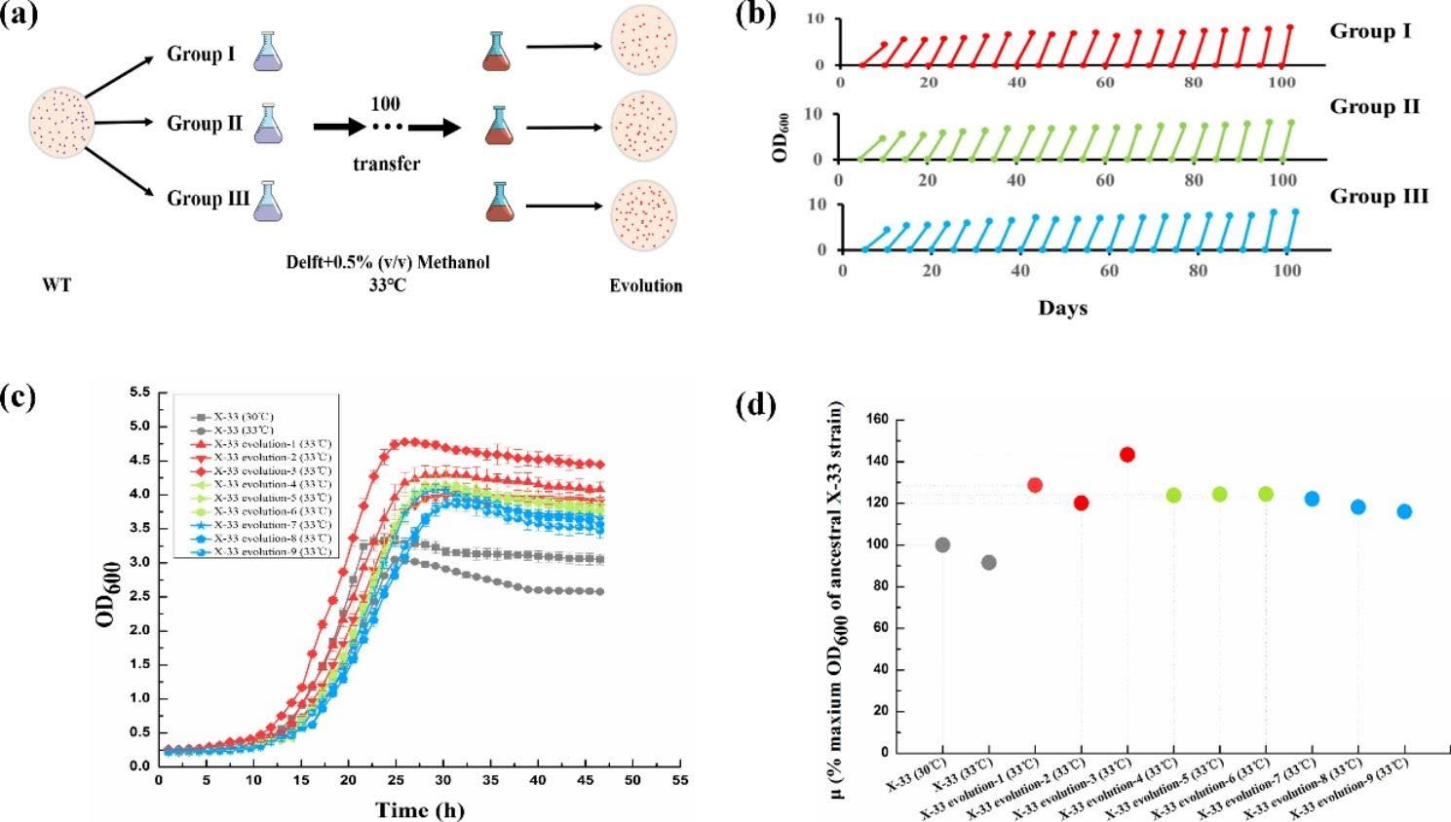
ALE enhanced final biomass and tolerance to 33 °C of *P. pastoris* in 0.5% methanol minimal medium. **a**, The process of ALE; **b**, Procedure of ALE that was illustrated by enhanced cell growth; **c**, Automatic growth curves of the starting strain X-33 at 30 or 33 °C and the evolved strains at 33 °C; **d**, % maximum OD_600_ of the starting strain X-33 and the evolved strains at 33 °C relative to the starting strain X-33 at 30 °C. Three independent colonies of X-33 were transferred to a 250 ml shake flask with 100 mL of 0.5% methanol minimal medium with an initial OD_600_ of 0.25 at 33 °C at 220 rpm. Transferred the evolved strains to fresh growth medium every 24 h, until a high density of cells were obtained. Final evolved strains were spotted on YPD plates for further growth analysis using an automatic microbial growth curve analyzer. Data are average values and standard deviations of triplicate experiments

To avoid methanol evaporation, ALE was performed in the shake flasks covered with a sealing membrane. The process of ALE is graphically explained in Fig. 2a. Briefly, parent strain *P. pastoris* X-33 was spotted on a YPD agar plate and three independent colonies were cultivated in a 10 ml glass tube with 3 ml of Delft basic salt medium containing 2% glycerol for 24 h. These three strains were transferred to a 250 ml shake flask with 100 ml of Delft basic salt medium containing 0.5% methanol with an initial OD_600_ of 0.25. After 24 h of cultivation at 33 °C in a rotatory shaker at 220 rpm, the strains were transferred to the fresh medium with the same initial OD_600_, which were then incubated under the same conditions. Repeated the above steps about 100 times until a high density of cells were obtained. The final evolved colonies were streaked on YPD plates, and three colonies were picked from each group for further cell growth analysis.

### Automatic microbial growth curve analysis

The cell growth of *P. pastoris* was evaluated using an automatic microbial growth curve analyzer. Briefly, overnight cultures of strains were inoculated into a 48-well plate containing 1 ml Delft basic salt medium containing 0.5% methanol with an initial OD_600_ of 0.25 per well at 30 or 33 °C at 800 rpm. Cell growth was measured every hour to plot the growth curve and calculate final biomass. Three replicates per condition were used.

### Transcriptome analysis

The control strain X-33 and the evolved strain HTX-33 were grown in Delft basic salt medium containing 0.5% methanol at 30 and 33 °C, respectively. The total RNA was extracted from the cells under these two culture conditions when grown to the exponential phase using TRIzol (Invitrogen, Carlsbad, CA, USA) in accordance with the manufacturer’s protocols. RNA with the integrity of more than 6.5 that was detected by Agilent 2100 Nano (Agilent Technologies) was adopted to perform library construction and sequencing. The complementary DNA libraries were constructed and sequenced using the Illumina HiSeq 2000 platform at the Meiji Biotechnology (Shanghai) Co., Ltd. RESM software (https://deweylab.github.io/RSEM/) was used to quantify gene expression levels, yielding transcripts per million reads (TPM). The differential expression between the two samples was analyzed by the DEGseq (https://bioinfo.au.tsinghua.edu.cn/software/degseq), and genes with |log2FC|≥1 and *P* < 0.05 were identified as significantly differentially expressed genes (DEGs). The Kyoto Encyclopedia of Genes and Genomes (KEGG) enrichment analysis was performed using the KOBAS (https://kobas.cbi.pku.edu.cn/home.do).

### Formaldehyde quantification

Cells grown to the mid-log phase from 48-well plates were harvested by centrifugation at 5000×*g* for 10 min, washed three times and resuspended in 1 mL of PBS buffer solution (pH = 7.4). Added equal volume of cell lysate and then incubated at 37 °C for 30 min. After centrifugation, the supernatants were collected for formaldehyde detection using the method as described previously [32]. Briefly, 50 µL of supernatant collected after the cultivation was added into a 96-well plate. 150 µL of NASH reagent (5 M ammonium acetate, 50 mM acetyl acetone and 135 mM acetic acid) were then added into each well. The plate was kept on ice until all samples had been added. Incubated the plate at 37 °C for 10 min and read the absorbance at 414 nm. The formaldehyde concentrations were expressed as µM/OD.

### Validation of genes by real-time quantitative polymerase chain reaction (RT-qPCR)

Twenty-one DEGs were selected to test the reliability of the RNA-Seq data by RT-qPCR. Total RNA was extracted from X-33 and HTX-33 strains grown to the exponential phase, respectively, using the TransZol Up Plus RNA Kit (TransGen, Beijing, China). The extracted RNA was then tested for its quality and concentration using a Nanodrop 2000c (Thermo). And cDNA was synthesized from 200 ng RNA using Quant Reverse Transcriptase in the presence of random primers (TransGen, Beijing, China). RT-qPCR was carried out using Real Master Mix (SYBR Green) and specific primers (Supplementary Table 2) in a Light Cycler 480 II (Roche, Basel, Switzerland) under the following cycling conditions: 5 min at 50 °C, 30 s at 94 °C, 45 cycles of 5 s at 94 °C, and 30 s at 60 °C. The *ACT1* gene was used as a reference gene for normalization and all reactions were carried out in triplicate. Relative transcription levels were analyzed using the 2^−ΔΔCt^ method described previously [33].

### Cell morphology analysis

Scanning electron microscopy (SEM) was used to visualize the changes in the cell wall surface of *P. pastoris*. Transmission electron microscopy (TEM) was used to check the thickness of the cell wall of *P. pastoris*. Cells grown to the mid-log phase were harvested by centrifugation at 5000×*g* for 10 min, washed three times, and resuspended in PBS buffer solution (pH = 7.4). The suspension was premixed with an equal volume of 2.5% glutaraldehyde for 12 h at 4◦C and subsequently dehydrated with 25, 50, 70, 80, 95, and 100% ethanol. For SEM, the dehydrated samples were air-dried immediately, followed by smearing on SEM stubs and gold covering. The micrographs of the cell wall surface were obtained using a SEM (Hitachi SU8010, Tokyo, Japan). For TEM, pure ethanol was changed to propylene oxide and specimens were gradually infiltrated with increasing concentrations (30, 50, 70, and 100%) of Agar 100 epoxy resin mixed with propylene oxide for a minimum of 3 h per step. Samples were embedded in pure, fresh Agar 100 epoxy resin and polymerized at 60 °C for 48 h. Ultrathin sections were stained for 3 min with lead citrate and viewed with a JEM-1230 electron microscope. As for observation of colony morphology, *P. pastoris* strains stored at -80◦C were streaked on YPD agar plates. After the strain was cultured at 30 or 33 °C for 72 h, the morphology of the colonies were observed and photographed with a digital camera.

### Biomass and total protein determination

Overnight cultures of *P. pastoris* strains were inoculated into a 48-well plate containing 1 mL 0.5% methanol minimal medium with an initial OD_600_ of 0.25 at 30 or 33 °C at 800 rpm. Cell pellets from 3 mL of mid-log phase culture were washed with sterile water three times and resuspended in 1 mL of sterile water. The tubes containing centrifuged cells were dried at 105 °C until a constant weight was maintained. The total nitrogen content was measured using the Kjeldahl method [34]. A conversion factor of 6.25 was used to convert nitrogen content to protein content. Total nitrogen content and protein content are proportional to yeast dry mass (%).

### Pilot-scale fed-batch fermentation and nutritional benefit assessment

Pilot-scale fed-batch fermentation was performed in a 500 L automatic bioreactor system (Shanghai Bailun Biotechnology Co., Ltd). The initial batch fermentation was carried out in Delft + 2% glycerol with 300 L working volume. The initial inoculum was 28 vials of overnight culture strain HTX-33-GLN1-Δ*PAS_chr4_0305* in Delft + 2% glycerol. Temperature and pH were set at 33 °C and 5.6, respectively. Initial agitation was set at 100 rpm/min and increased to a maximum of 300 rpm/min depending on the air volume level. Air volume was initially provided at 0.55 vvm and increased to a maximum of 1.5 vvm. Fed-batch cultivation started immediately after the glycerol in the batch medium was exhausted and methanol was pumped into the vessel. During fermentation, residual methanol was monitored to control the feeding rates, and biomass and methanol consumption were recorded every 2 h. After fermentation, all cells were centrifuged, washed and dried. The finished products were used for subsequent total protein determination as mentioned above and amino acids profile analysis through an automatic amino acid analyzer. Total protein content and amino acids content are related to yeast dry mass (%).

### Statistical analysis

One-way analysis of variance was implemented in SPSS for Windows 10.0 (SPSS, Inc., Chicago, IL, United States).

## Results

### Adaptive laboratory evolution towards growth on methanol and higher temperature

ALE is recognized as a powerful strategy for achieving desirable phenotypes via causal mutations [11, 17, 35], and was thus used in this study to promote the growth of X-33 strain in methanol minimal medium at higher temperature. ALE was performed in Delft basic salt medium containing 0.5% methanol at 33 °C at 220 rpm, and three independent colonies of the X-33 strain were adopted as the starting strain, which was subsequently transferred to fresh growth medium in 24-h intervals for 100 days, yielding a total of approximately 350 generations. As shown in Fig. 2b, all three groups acquired higher cell growth in a minimal medium containing 0.5% methanol at 33 °C. Three colonies were randomly selected from each group to characterize their growth performance using an automatic microbial growth curve analyzer. As can be seen in Fig. 2c, the final biomass of strain X-33 at 33 °C (up to OD_600_ of 3.05) was 91.59% of that at 30 °C (up to OD_600_ of 3.33), indicating that higher temperature inhibited the cell growth of *P. pastoris*. All of nine clones from the three evolved populations showed increased final biomass on this growth medium, of which the highest final biomass (up to OD_600_ of 4.78) accounted for 1.43 times that of the starting strain X-33 cultured at 30 °C (Fig. 2d). Finally, this superior-performance strain with high conversion efficiency of methanol to biomass and 33 °C tolerance was named HTX-33.

### Changes in cell wall and protein content of the evolved strain HTX-33

**Fig. 3:**
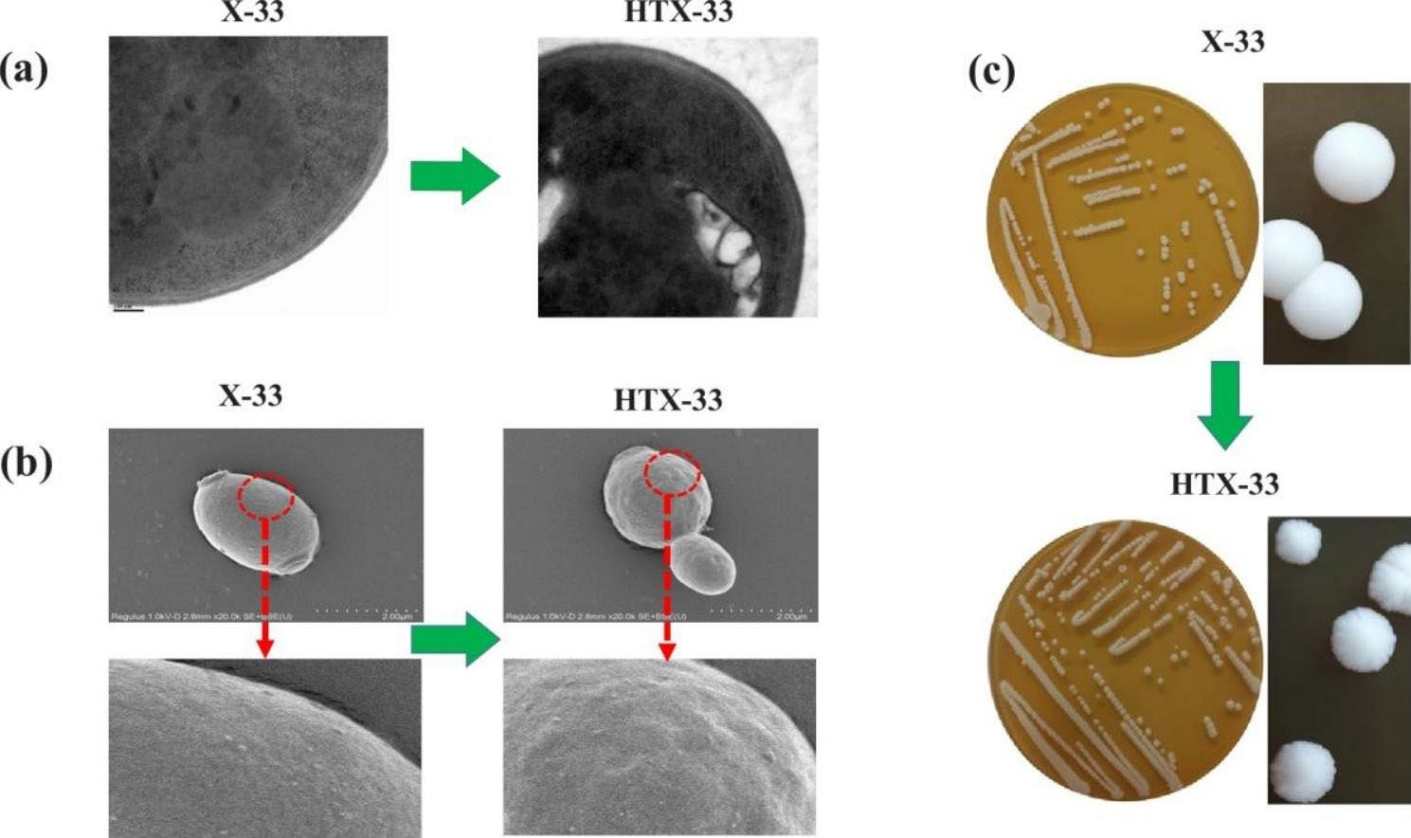
Changes in cell wall and colony morphology of starting strain X-33 at 30 °C and the evolved strain HTX-33 at 33 °C in 0.5% methanol minimal medium. **a**, Representative TEM images of cell wall thickness; **b**, Representative SEM images of cell wall surface. The surface of the cell wall is partially magnified; **c**, Changes in colony morphology. Representative images are from three independent experiments

The cell wall is the toughest outer layer of yeast cells and is important for supporting cell structure and protecting cells from chemical and physical damage [36]. Since the cell wall is the first line of defense against external stress, cell wall strength and efficient cell wall remodeling are essential to withstand higher temperature stress. Studies have shown that genes involved in cell wall biogenesis were altered after cells evolved in a high temperature environment, resulting in a strengthened cell wall [37]. Consistent with this finding, the cell wall thickness of the evolved strain HTX-33 was increased after adaptation at 33 °C (Fig. 3a). Furthermore, SEM results showed that the cell wall surface of HTX-33 strain had obvious wrinkles, which was in strong contrast with the relatively smooth surface of X-33 (Fig. 3b). These changes in cell wall structure eventually led to the irregular round shape of the HTX-33 colonies (Fig. 3c). In addition, it was found that the total nitrogen (g/g DCW) and protein content (g/g DCW) of the X-33 strain at 33 °C (0.064 and 0.4, respectively) were 10% higher than those at 30 °C (0.058 and 0.364, respectively) (Table 2), which might be beneficial for tolerance to higher temperature due to increased protein synthesis. However, the total nitrogen and protein content of evolved strain HTX-33 were decreased to 0.059 and 0.368, respectively (Table 2), after long-term evolution at 33 °C. Our results indicated that the cell wall was strengthened in response to higher temperature stress, but the protein content was not further improved in the evolved strain HTX-33 compared to the starting strain X-33 at 30 °C.

**Table 2:** Changes in total nitrogen and protein content of different *P. pastoris* strains in 0.5% methanol minimal medium

Strains	Total nitrogen (g/g DCW)	Protein content (g/g DCW)	Increased % ratio compared with X-33 at 30 °C
X-33 (30 °C)	0.058 ± 0.004	0.364 ± 0.028	0
X-33 (33 °C)	0.064 ± 0.002^*^	0.400 ± 0.012^*^	10%
HTX-33	0.059 ± 0.002	0.368 ± 0.016	1%
HTX-33-GDH1	0.063 ± 0.003^*^	0.394 ± 0.016^*^	8%
HTX-33-GLN1	0.066 ± 0.003^*^	0.414 ± 0.009^*^	14%
HTX-33-GDH1-GLN1	0.065 ± 0.001^*^	0.413 ± 0.007^*^	13%
HTX-33-Δ*PAS_chr4_0305*	0.071 ± 0.002^**^	0.444 ± 0.012^**^	22%
HTX-33-GLN1-Δ*PAS_chr4_0305*	0.073 ± 0.004^***^	0.456 ± 0.025^***^	25%

Unless otherwise specified, *P. pastoris* strains were cultivated in 48-well plates containing 0.5% methanol minimal medium at 33 °C at 800 rpm. The total nitrogen and protein content of *P. pastoris* strains were determined when grown to the exponential phase. Data are average values and standard deviations of triplicate experiments. An asterisk indicates a significant difference with ^*^P < 0.05, ^**^P < 0.01, ^***^P < 0.001

### Transcriptomics analysis and intracellular formaldehyde determination of the evolved strain HTX-33

**Fig. 4:**
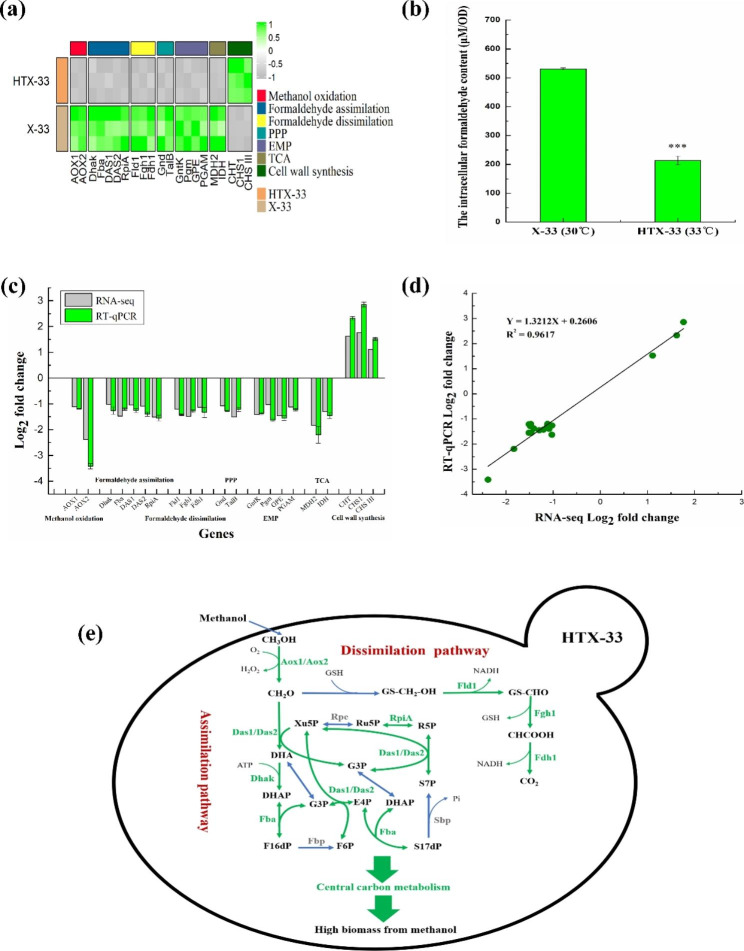
DEGs and intracellular formaldehyde accumulation analyses of the evolved strain HTX-33 at 33 °C compared to the starting strain X-33 at 30 °C in 0.5% methanol minimal medium. **a**, Cluster analysis related to DEGs from methanol metabolism, PPP, EMP, TCA and cell wall synthesis; **b**, Changes in intracellular formaldehyde accumulation (µM per OD); **c**, Validation of selected DEGs by RT-qPCR; **d**, Correlation of the RNA-Seq data (X-axis) and the RT-qPCR data (Y-axis). Data are average values and standard deviations of triplicate experiments. An asterisk indicates a significant difference with ^***^P < 0.001; **e**, Sketch of DEGs in methanol metabolism. Genes in green showed significant down-regulation in expression. Bold border represented enhanced cell wall. Enzymes: Aox, alcohol oxidase; Fld1, formaldehyde dehydrogenase; Fgh1, S-formylglutathione hydrolase; Fdh1, formate dehydrogenase; Das, dihydroxyacetone synthase; Dhak, dihydroxyacetone kinase; Fba, fructosebisphosphate aldolase/sedoheptulose-bisphosphate aldolase; Fbp, fructose bisphosphatase; Sbp, sedoheptulose bisphosphatase; Rpe, ribulose-phosphate 3-epimerase; RpiA, ribose-5-phosphate isomerase; Metabolites: Xu5P, xylulose-5-phosphate; Ru5P, ribulose-5-phosphate; R5P, ribose-5-phosphate; DHA, dihydroxyacetone; DHAP, dihydroxyacetone phosphate; F16dP, fructose-1,6-bisphosphate; F6P, fructose-6-phosphate; G3P, glyceraldehyde-3-phosphate; E4P, erythrose-4-phosphate; S7P, sedoheptulose-7-phosphate; S17dP, sedoheptulose-1,7-bisphosphate

Subsequently, the whole-transcriptome analysis was conducted to identify essential DEGs related to methanol utilization and higher temperature stress in the evolved strain HTX-33 at 33 °C relative to the parent strain X-33 at 30 °C in minimal medium containing 0.5% methanol. The RNA-Seq data revealed that for the evolved strain HTX-33, 592 genes were significantly differentially expressed, of which 293 were upregulated and 299 were downregulated (Supplementary Table 3). The KEGG enrichment analysis showed that a total of 87 pathways had been enriched, and only methane metabolism (also regarded as methanol metabolism) pathway was significantly enriched (*P* < 0.05) (Supplementary Table 4). Unexpectedly, genes in methanol oxidation including *PAS_chr4_0821* (encoding AOX1, alcohol oxidase 1) and *PAS_chr4_0152* (encoding AOX2, alcohol oxidase 2), formaldehyde assimilation including *PAS_chr3_0841* (encoding Dhak, dihydroxyacetone kinase), *PAS_chr1-1_0319* (encoding Fba, fructose 1,6-bisphosphate aldolase), *PAS_chr3_0834* (encoding DAS1, dihydroxyacetone synthase 1), *PAS_chr3_0832* (encoding DAS2, dihydroxyacetone synthase 2) and *PAS_chr4_0212* (encoding RpiA, ribose-5-phosphate ketol-isomerase), and formaldehyde dissimilation including *PAS_chr3_1028* (encoding Fld1, S-(hydroxymethyl) glutathione dehydrogenase), *PAS_chr3_0867* (encoding Fgh1, S-formylglutathione hydrolase) and *PAS_chr3_0932* (encoding Fdh1, NAD^+^-dependent formate dehydrogenase) were greatly downregulated for the higher growth of evolved strain HTX-33 in methanol minimal medium at 33 °C (Fig. 4a). Since formaldehyde toxicity is supposed to be one of the primary obstacles limiting the carbon loss encountered by *P. pastoris* [27], the intracellular formaldehyde accumulation was characterized in the control strain X-33 and the evolved strain HTX-33. As expected, the HTX-33 strain accumulated a low level of formaldehyde at 214 µM/OD, compared to a relatively high level of formaldehyde in X-33 cells up to 531 µM/OD within 24 h (Fig. 4b).

In addition, genes involved in pentose phosphate pathway (PPP) including *PAS_chr3_0277* (encoding Gnd, 6-phosphogluconate dehydrogenase) and *PAS_chr2-2_0338* (encoding TalB, transaldolase), glycolysis pathway (EMP) including *PAS_chr1-4_0669* (encoding GntK, gluconokinase), *PAS_chr2-1_0771* (encoding Pgm, phosphoglucomutase), *PAS_chr1-4_0042* (encoding GPE, glucose-6-phosphate 1-epimerase) and *PAS_chr3_0693* (encoding PGAM, tetrameric phosphoglycerate mutase), TCA cycle including *PAS_chr4_0815* (encoding MDH2, mitochondrial malate dehydrogenase) and *PAS_chr2-1_0580* (encoding IDH, cytosolic NADP-specific isocitrate dehydrogenase) were also downregulated (Fig. 4a).

In contrast to most downregulated genes in methanol and central carbon metabolism, genes participated in cell wall synthesis including *PAS_chr4_0559* (encoding CHT, putative chitin transglycosidase, cell wall protein), *PAS_chr1-1_0393* (encoding CHS1, Chitin synthase I) and *PAS_chr2-1_0065* (encoding CHS III, Chitin synthase III, catalyzes the transfer of N-acetylglucosamine to chitin) were upregulated (Fig. 4a), which may help to explain the changes in cell wall of the evolved strain HTX-33, thereby endowing the strain with higher temperature tolerance.

Based on the above analysis, seven sets of genes related to methanol oxidation, formaldehyde assimilation, formaldehyde dissimilation, PPP, EMP, TCA and cell wall synthesis were then selected for RT-qPCR analysis to verify the accuracy and reproducibility of RNA-Seq data. As expected, the expression trends of the 21 candidate DEGs were consistent with those detected by RNA-seq (Fig. 4c), and the correlation coefficient between RNA-Seq and RT-qPCR data was 0.9617 (Fig. 4d), indicating that the RNA-Seq data are accurate and reliable. In summary, although methanol and central carbon metabolism were slowed down, the reduction in carbon loss due to the lessened detoxification of intracellular formaldehyde through the dissimilation pathway ultimately increased methanol utilization efficiency (Fig. 4e). Further, it was also found that the ability to effectively induce cell wall strengthening is necessary for protecting cells against higher temperature stress in the evolved strain HTX-33 (Fig. 4e).

### Enhancement of protein content by regulating nitrogen metabolism

**Fig. 5:**
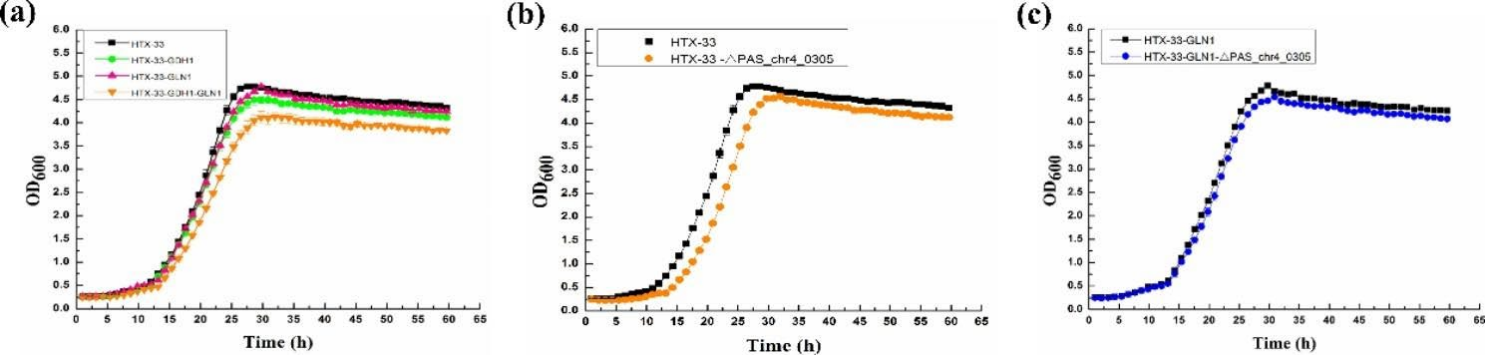
Effects of regulating nitrogen metabolism and engineering cell wall on cell growth of *P. pastoris* in 0.5% methanol minimal medium at 33 °C. **a**, Effects of overexpression of GDH1 and GLN1 on cell growth of strain HTX-33; **b**, Effect of deletion of *PAS_chr4_0305* on cell growth of strain HTX-33; **c**, Effect of deletion of *PAS_chr4_0305* on cell growth of strain HTX-33-GLN1. Data are average values and standard deviations of triplicate experiments

Even though ALE solved the problem of low methanol utilization efficiency and intolerance to higher temperature, the protein content of the evolved strain HTX-33 remains low, which requires further regulation. Nitrogen metabolism has been shown to be critical for the catabolism and anabolism of proteins. In yeast, nitrogenous substances for amino acid biosynthesis are mainly converted from glutamate and glutamine. Amino nitrogen from glutamate and amide from glutamine account for 85% and 15% of total cellular nitrogen, respectively [38]. Glutamate is synthesized mainly by a reaction between-ketoglutarate and ammonia, which is catalyzed by NADPH-dependent glutamate dehydrogenase (GDH1) [39], and glutamate could further be used to synthesize glutamine with ammonia, which is catalyzed by glutamine synthase (GLN1) [40, 41]. Glutamate and glutamine, together with α-ketoglutarate, link the TCA cycle and nitrogen metabolism [42]. This study attempted to improve the protein content of strain HTX-33 by regulating nitrogen metabolism through overexpression of GDH1 and GLN1. It was found that overexpression of GDH1 in HTX-33 reduced final biomass to 4.49 (Fig. 5a), while the total nitrogen and protein content was increased to 0.063 and 0.394 (representing 8% increase compared with X-33 at 30 °C) (Table 2), respectively, when cultured in 0.5% methanol minimal medium at 33 °C. Notably, overexpression of GLN1 in HTX-33 increased total nitrogen to 0.066 and protein content to 0.414 (representing 14% increase compared with X-33 at 30 °C) (Table 2), respectively, without reducing final biomass (Fig. 5a). However, simultaneous overexpression of GDH1 and GLN1 resulted in a decrease in final biomass (Fig. 5a), and the increase in total nitrogen and protein content did not have a significant advantage over overexpression of GLN1 alone (Table 2). The above results indicate that overexpression of GDH1 and GLN1 in the nitrogen metabolism pathway could increase the protein content in the evolved strain HTX-33 at 33 °C, and that the conversion of methanol to biomass remains the same as in HTX-33 with GLN1 overexpression alone.

### Enhancement of protein content by impairing cell wall synthesis

**Fig. 6:**
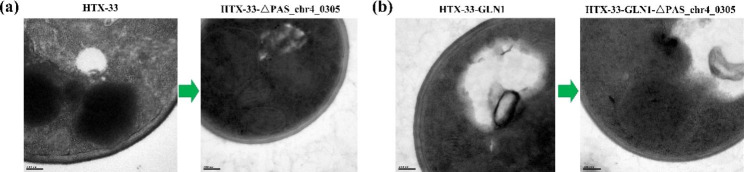
Effects of deletion of *PAS_chr4_0305* on the cell wall thickness of *P. pastoris* in 0.5% methanol minimal medium at 33 °C. **a**, Representative TEM images after deletion of *PAS_chr4_0305* in strain HTX-33; **b**, Representative TEM images after deletion of *PAS_chr4_0305* in strain HTX-33-GLN1. Representative images are from three independent experiments

In a previous study, we discovered that as methanol concentration increased (from 0.5 to 3%), the cell wall thickened, resulting in a significant decrease in protein content in *P. pastoris*. Further RNA-Seq results showed that the upregulation of the gene *PAS_chr4_0305*, encoding the protein involved in *O*-glycosylation required for cell wall stability, predominated in promoting cell wall synthesis in response to the increase in methanol concentration (data not shown). These results led us to infer that impairing cell wall synthesis by knocking out the gene *PAS_chr4_0305* may be an efficient engineering strategy to increase the protein content of *P. pastoris*. Here, deletion of gene *PAS_chr4_0305* was performed in the evolved strain HTX-33 at 33 °C. In response to this modification, the cell wall thickness was reduced (Fig. 6a), and correspondingly, the total nitrogen and the protein content were increased to 0.071 and 0.444, respectively, which increased by 22% compared with X-33 at 30 °C (Table 2); however, the final biomass was slightly reduced (Fig. 5b). These results indicate that although the inactivation of gene *PAS_chr4_0305* increased the sensitivity of the strain to higher temperature, resulting in a slight decrease in the conversion of methanol to biomass at 33 °C, the disruption of cell wall synthesis could still improve the protein content of evolved strain HTX-33 under this culture condition.

Further, the gene *PAS_chr4_0305* was deleted in strain HTX-33-GLN1, resulting in strain HTX-33-GLN1-Δ*PAS_chr4_0305*. Likewise, the cell wall thickness was decreased (Fig. 6b), and correspondingly, the total nitrogen and protein content of strain HTX-33-GLN1-Δ*PAS_chr4_0305* at 33 °C reached 0.073 and 0.456, respectively, which were 25% higher than those of the wild-type strain X-33 at 30 °C (Table 2). In addition, although the deletion of the gene *PAS_chr4_0305* reduced the conversion of methanol to biomass to a certain extent, the final biomass of the strain HTX-33-GLN1-Δ*PAS_chr4_0305* could reach 4.54 at 33 °C, which was 1.36 times that of the wild-type strain X-33 at 30 °C (Figs. 2c and 5c). Thus, a *P. pastoris* strain HTX-33-GLN1-Δ*PAS_chr4_0305* with high methanol utilization, tolerance to 33 °C and high protein synthesis was obtained through the combination of ALE, strengthening nitrogen metabolism and impairing cell wall synthesis.

### Pilot-scale fed-batch fermentation

**Fig. 7:**
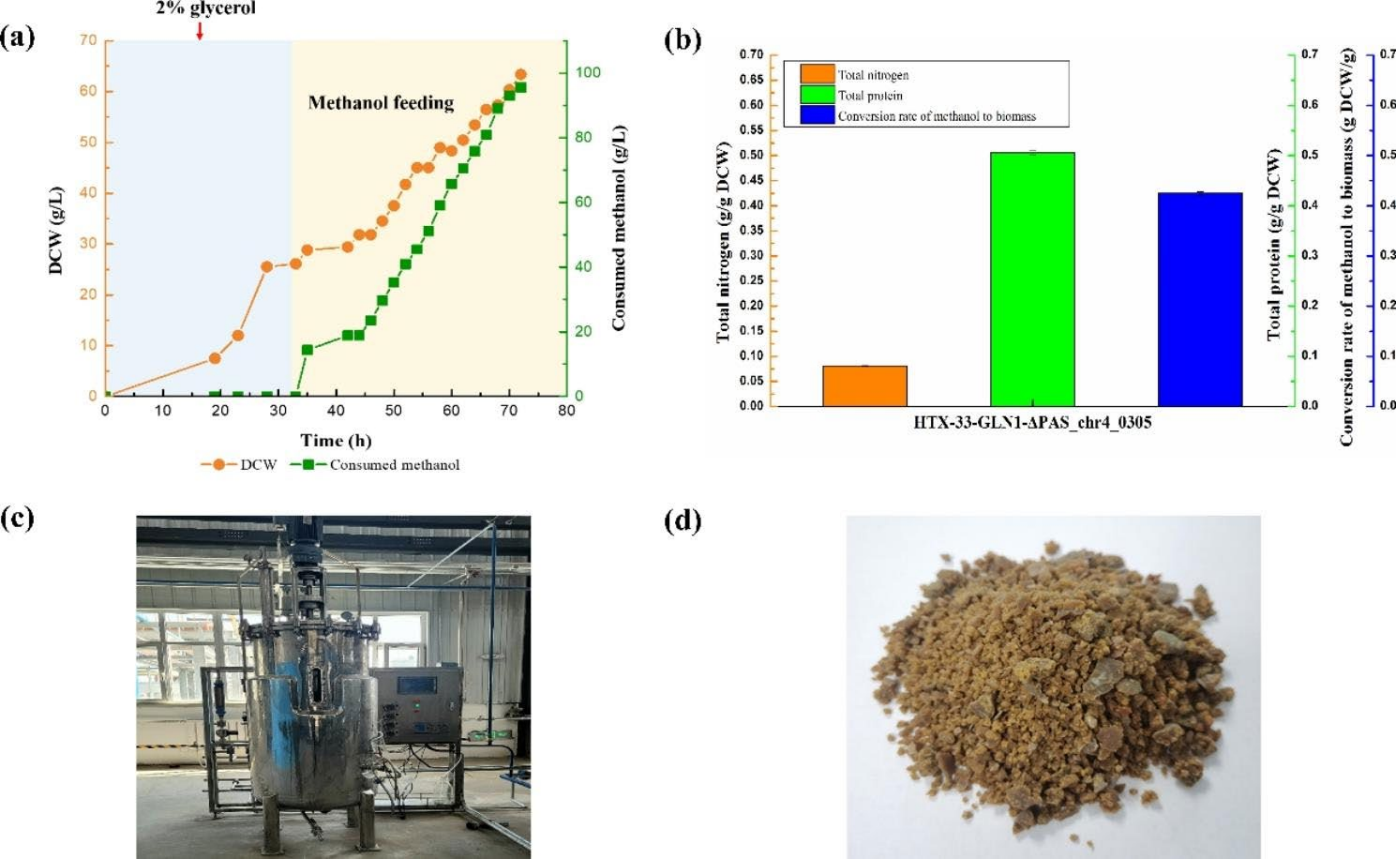
Pilot-scale fed-batch fermentation of strain HTX-33-GLN1-Δ*PAS_chr4_0305* in bioreactor. **a**, Changes in biomass and methanol consumption. Pilot-scale fed-batch fermentation was performed in a 500 L automatic bioreactor system with 300 L working volume. Temperature and pH were set at 33 °C and 5.6, respectively. Fed-batch fermentation was carried out with 2% glycerol in the initial batch stage. Methanol feeding was started at 33 h, achieving the maximum biomass at 72 h; **b**, The total nitrogen, the protein content and the conversion rate of methanol to biomass of strain HTX-33-GLN1-Δ*PAS_chr4_0305* in the bioreactor; **c**, The pilot-scale bioreactor used in this experiment; **d**, *P. pastoris* SCP sample after centrifugation, washing and drying. Data are average values and standard deviations of triplicate experiments

To test the industrial potential of our engineered strain to produce SCP from methanol at 33 °C, we performed fed-batch fermentation in a 500 L (the filling volume was 300 L) bioreactor using the best strain HTX-33-GLN1-Δ*PAS_chr4_0305*. Seed liquor was obtained by fed-batch fermentation with 2% glycerol in the initial batch stage. The methanol feed started at 33 h, the methanol induction stage was at 33 to 35 h, and the fermentation stage was at 35 to 72 h. The bioreactor process stopped at 72 h due to the large amount of heat generated during *P. pastoris* growth. In the fermentation process, the highest levels of biomass (DCW) reached 63.37 g/L with a methanol conversion rate of 0.43 g DCW/g, amounting to 86% of the maximum theoretical yield (0.5 g DCW/g). Interestingly, the total nitrogen and the protein content from bioreactor-cultured cells reached 0.081 g/g DCW and 0.506 g/g DCW, respectively, which were much higher than those of well plate cells due to the more abundant supply of oxygen in the bioreactor (Fig. 7). Based on the above data and the cost accounting (Supplementary Table 5), the conversion rate of methanol to protein can be converted to 0.22 g/g and the cost of SCP was caculated as 29.24 CNY/kg DCW in the bioreactor. All the above findings indicate the outstanding potential of our engineered strain for economical industrial application using methanol as a feedstock for SCP production at 33 °C.

### Nutritional benefits of SCP from ***P. pastoris***

**Fig. 8:**
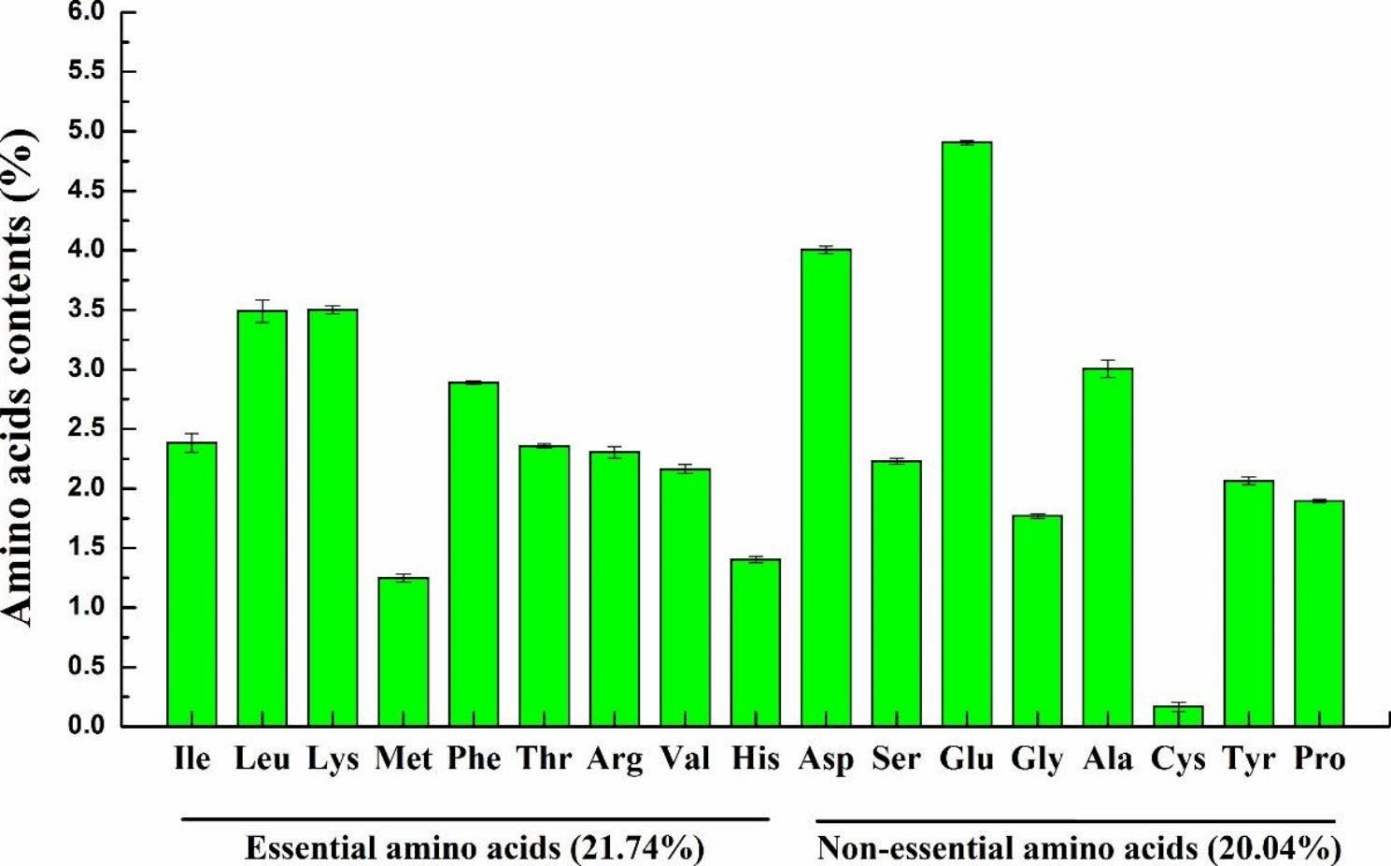
The amino acids profile of SCP produced by strain HTX-33-GLN1-Δ*PAS_chr4_0305* cultured in the bioreactor. Ile, isoleucine; Leu, leucine; Lys, lysine; Met, methionine; Phe, phenylalanine; Thr, threonine; Arg, arginine; Val, valine; His, histidine; Asp, aspartic acid; Ser, serine; Glu, glutamic acid; Gly, glycine; Ala, alanine; Cys, cystine acid; Tyr, tyrosine; Pro, proline. Data are average values and standard deviations of triplicate experiments

In addition to protein content, the amino acid composition is also a key determinant of SCP quality [2]. In this study, the amino acids profile of SCP produced by strain HTX-33-GLN1-Δ*PAS_chr4_0305* cultured in the bioreactor were quantified. As shown in Fig. 8, a total of 17 amino acids were detected, accounting for 41.78% of DCW, of which 21.74% were essential amino acids, including 2.38% isoleucine, 3.49% leucine, 3.5% lysine, 1.25% methionine, 2.89% phenylalanine, 2.36% threonine, 2.31% arginine, 2.16% valine and 1.4% histidine. It should be noting that SCP from *P. pastoris* is a source of the limiting amino acids lysine and methionine, of which the former is deficient in cereals, and the latter is relatively deficient in soy, peanut, milk and meat proteins [2, 43–45], suggesting that SCP from *P. pastoris* could be mixed to obtain a more nutritive food. Another interesting finding is that the total amount of branched-chain amino acids (BCAAs: valine, isoleucine, leucine) is up to 8.03%. BCAAs are important energy source in the body, which could increase ATP production by promoting glucose uptake [46]. BCAAs are also involved in the regulation of body lipid metabolism, protein synthesis and immune response etc., [47, 48]. Thus, SCP from *P. pastoris* is also a source of BCAAs supplements in low-protein foods. In summary, SCP from *P. pastoris* (50.6% in this report) generally contains a higher percentage of protein compared to soy (38.6%), fish (17.8%), meat (21.2%) and whole milk (3.28%) [2]. SCP from *P. pastoris* is a source of essential amino acids, including methionine, lysine, and BCAAs.

## Discussion

SCP biosynthesis from *P. pastoris* using C1 feedstock offers an attractive alternative to animal-derived proteins due to its rapid production rate, lower space requirements, independence of climate or seasons and more sustainable production process [1, 2, 8]. Nowadays, some companies (Such as Calysta, DeepBranch etc.) have successfully produced SCP by CO_2_ and methane, but most of these processes are anaerobic fermentation, which is inefficient and ultimately low in biomass. On the contrary, the production of SCP by *P. pastoris* is an aerobic fermentation process with high production efficiency and high biomass. Methanol is an ideal C1 feedstock for biomanufacturing due to its high reduction potential and extensive production sources [12–14]. At present, the methanol production by CO_2_ and hydrogen has been widely recognized [49]. The cost of the methanol production via CO_2_ and hydrogen is determined by the cost of electricity. As electricity gets cheaper due to the development of renewable energy, the methanol is also getting cheaper. In order to achieve the carbon neutrality goal for the entire planet, large-scale biomanufacturing based on methanol may contribute to the creation of process chains leading to SCP with a (nearly) zero CO_2_ footprint. However, the complexity of methanol metabolism pathway and the toxicity of intracellular formaldehyde hinder the efficient utilization of methanol in *P. pastoris* [27, 28]. Improving methanol utilization is of great significance to the economical industrial application using methanol as a feedstock for SCP production. It is true that using *P. pastoris* to SCP production at higher temperature could be not only adapt to the industrial environment in summer, but could also decrease the cooling water consumption. However, the cell wall will be strengthened in response to higher temperature stress [37], which is not conducive to the synthesis of protein in *P. pastoris* as found in this study. After all, the nutritional value of SCP is closely related to the protein content of *P. pastoris*, a *P. pastoris* strain with high-protein content will be more competitive with other protein sources [2]. Furthermore, higher temperature could inhibit the cell growth of *P. pastoris* in methanol minimal medium. Our preliminary research shows that *P. pastoris* can grow well at 28 and 30 °C. The cell growth was lightly repressed at 33 °C but strongly repressed at 35 °C. Notly, *P. pastoris* can not grow at 37 °C in 0.5% methanol minimal medium (Supplementary Fig. 1) [50]. In consideration of both biomass and protein content, we finally choose a higher temperature of 33 °C as an evolution pressure. Using *P. pastoris* to SCP production at 33 °C still have the potential in reducing production costs. In this study, the optimal evolved strain HTX-33 achieved the highest final biomass in 0.5% methanol minimal medium cultured at 33 °C, representing 1.43 times that of the parent strain X-33 cultured at 30 °C, indicating that both the low methanol utilization efficiency and the inability to tolerate higher temperature were solved through adaptive evolution.

Furthermore, transcriptomic analysis revealed that the majority of genes involved in methanol metabolism, such as alcohol oxidase genes *AOX1* and *AOX2*, formaldehyde assimilation key genes *DAS1* and *DAS2*, and formaldehyde dissimilation genes *Fld1*, *Fgh1* and *Fdh1*, were all significantly downregulated in the evolved strain HTX-33. A long-term evolution of strain HTX-33 resulted in a reduced accumulation of intracellular formaldehyde. In fact, we performed genetic sequencing of all DEGs involved in methanol metabolism and discovered that only the 1257th base of AOX1 was mutated (base C to base A), resulting in the 419th amino acid of AOX1 changing from F to L (date not shown). AOX1 is the first enzyme in the methanol utilization pathway, so it is not surprising that this key enzyme represents a potential selective target during the adaptation towards growth on methanol. The same mutation was also found in a previous report that long-term adaptation resulted in the selection of clones with mutations in the *AOX1* gene, which subsequently reduced alcohol oxidase activity and increased growth rates as compared with the ancestor [35]. Until now, the relationship among AOX1 activity, intracellular formaldehyde toxicity and methanol utilization efficiency has not been clearly elucidated. By combining the results of this study with previous findings, we concluded that reduced AOX1 activity decreased intracellular formaldehyde accumulation, which ultimately resulted in a reduction in carbon loss. Taken together, although the methanol and central carbon metabolism were slowed down after long-term evolution, the reduced carbon loss through the dissimilation pathway ultimately promoted the conversion of methanol to biomass in the evolved strain HTX-33. In addition, our results also revealed the effects of higher temperature on inducing cell wall stress. Consistent with previous findings [37], the cell wall thickening was observed after adaptive evolution at 33 °C, this may be a direct result of up-regulation of genes involved in cell wall synthesis. According to these results, *P. pastoris* cells require the ability to induce cell wall remodeling to withstand the stress associated with higher temperature during fermentation.

Besides being able to withstand higher temperature and reduce carbon loss, a *P. pastoris* strain with high protein content will necessarily be able to produce methanol-based SCP at a competitive level [2]. This study identified that overexpression of GDH1 or GLN1 in the nitrogen metabolism pathway could promote protein content in the evolved strain HTX-33 at 33 °C. Overexpression of GDH1 increased the protein content at the expense of reduced biomass, which may be a consequence of the disruption of the reducing equivalent of NADPH following overexpression of NADPH-dependent GDH1. Fortunately, overexpression of GLN1 increased protein content without reducing biomass. However, simultaneous overexpression of GDH1 and GLN1 did not have significant advantage over overexpression of GLN1 alone in terms of cell biomass and protein content in evolved strain HTX-33 at 33 °C. Knowledge gained from *P. pastoris* nitrogen metabolism pathway engineering indicates that overexpressing glutamine synthase GLN1 is a potential strategy to increase protein content of *P. pastoris* in methanol medium. So far, there was few studies adapted *P. pastoris* cell wall engineering for enhancing protein content. In yeast, most of the cell wall components are polysaccharides, and only a very small part is protein [36]. Consequently, it is reasonable to infer that impairing cell wall synthesis could be an effective strategy to reduce the C/N ratio, resulting in increase in protein content of *P. pastoris*. In this study, *PAS_chr4_0305*, a gene encoding the *O*-glycosylation related protein of cell wall, was found to be a key target for improving the protein content of *P. pastoris* on methanol as the sole carbon source. Although the knockout of gene *PAS_chr4_0305* increased the sensitivity of *P. pastoris* to higher temperature, the cell wall engineering by deletion of gene *PAS_chr4_0305* still increased the protein content of the evolved strain HTX-33 at 33 °C.

Finally, both overexpression of GLN1 and knockout of gene *PAS_chr4_0305* were implemented into the evolved strain HTX-33, creating the best strain HTX-33-GLN1-Δ*PAS_chr4_0305*, which achieved high-level production of SCP from sole methanol with a biomass of 63.37 g DCW/L, methanol conversion rate of 0.43 g DCW/g, and protein content of 0.506 g/g DCW in pilot-scale fed-batch culture at 33 °C. Indeed, the Phillips Petroleum Company has reported the 130 g/L of *P. pastoris* biomass on methanol during the 1970s. However, this high biomass was obtained based on the utilization of high-cell density cultivation method [29]. As we all known, the *P. pastoris* cells will grow slowly and most of methanol will be dissipated rather than utilized by the end of fermentation under high-density culture mode, which is not conducive to the economic production of SCP from methanol. Our research focuses on creating a *P. pastoris* strain with high methanol utilization, higher temperature resistance and high protein content, showing outstanding potential for economical industrial application using methanol as a feedstock for SCP production in the continuous fermentation mode. In this mode, we can continuously release the fermentation broth and feed at the stage of the highest methanol conversion rate, thus achieving continuous production of SCP without impairing the specific growth rate of *P. pastoris* strain. The protein content of SCP from *P. pastoris* reported here (50.6%) is much higher than that of soy (38.6%), fish (17.8%), meat (21.2%) and whole milk (3.28%) [2, 51]. In addition, compared with other SCP from low-value raw materials (Table 3), the protein content of SCP from *P. pastoris* reported here was almost equivalent to that of other fungi (11–63%) [52–56] and algae (42-64.9%) [5, 44, 57, 58], and slightly inferior to that of bacteria (41–83%) [59–62]. Further amino acid analysis showed that SCP from *P. pastoris* is a source of limiting amino acids, including lysine, methionine, and BCAAs supplements, indicating that SCP from *P. pastoris* could be mixed to obtain a more nutritive food. Future research could focus on fine-tuning the ratio of certain amino acids to increase the nutritional value of SCP from *P. pastoris*.

**Table 3:** Comparison of protein content with other SCP from low-value raw materials

Strains	Substrate	Protein content (%)	Reference
**Fungi**			
*Aspergilus niger*	Rice bran	11	(52)
*S. cerevisiae*	Food	40.2	(53)
*S. cerevisiae*	Food wastes	47.7	(54)
*A. niger*	Waste liquor	50	(55)
*P. pastoris*	Methanol	50.6	This study
*Pleurotus florida*	Wheat straw	63	(56)
**Algae**			
*Aphanothece microscopica*	CO_2_ (or bicarbonate) and light	42	(57)
*Chlorella pyrenoidosa*	CO_2_ (or bicarbonate) and light	45	(58)
*Chlorella* sp.	Food processing wastes (tofu)	52.3	(59)
*Haematococcus pluvialis*	Synthetic brewery wastewater	64.9	(60)
**Bacteria**			
*Methylomonas* and *Methylophilus*	Methane	41	(61)
*Cupriavidus necator* H16	Syngas (CO:H_2_ ratios at 1)	50.03	(62)
*Rhodopseudomonas faecalis*	Sugar industry wastewater	51.5	(63)
*Clostridium autoethanogenum* CICC 11088s	CO	83	(64)

## Conclusions

In summary, a *P. pastoris* strain with high methanol utilization efficiency, tolerance to 33 °C and high protein content was obtained through a combination of ALE, strengthening nitrogen metabolism and engineering the cell wall, showing outstanding potential for economical industrial application using methanol as a substrate for SCP production. SCP obtained from *P. pastoris* can have a higher protein content compared to conventional foods like soy, meat, milk and fish, and is a source of essential amino acids. SCP from *P. pastoris* using C1 feedstock is a promising alternative to obtain protein resource with environmental, economic and nutritional benefits, which is expected to become a sustainable production mode in the future.

### Electronic supplementary material

Below is the link to the electronic supplementary material.

Supplementary Material 1

Supplementary Material 2

Supplementary Material 3

Supplementary Material 4

Supplementary Material 5

Supplementary Material 6

## Data Availability

The data supporting the conclusions of this article are all available in the manuscript and supplementary.

## Abbreviations

C1: One-carbon
SCP: Single cell protein
ALE: Adaptive laboratory evolution
DCW: Dry cell weight
BCAAs: Branched-chain amino acids
GRAS: Generally recognized as safe
DEGs: Differentially expressed genes
KEGG: Kyoto encyclopedia of genes and genomes
SEM: Scanning electron microscopy
TEM: Transmission electron microscopy
RT-qPCR: Real-time quantitative polymerase chain reaction
TCA: Reductive tricarboxylic acid
PPP: Pentose phosphate pathway
EMP: Glycolysis pathway
Aox: Alcohol oxidase
Fld1: Formaldehyde dehydrogenase
Fgh1: S-formylglutathione hydrolase
Fdh1: Formate dehydrogenase
Das: Dihydroxyacetone synthase
Dhak: Dihydroxyacetone kinase
Fba: Fructosebisphosphate aldolase/sedoheptulose-bisphosphate aldolase
Fbp: Fructose bisphosphatase
Sbp: Sedoheptulose bisphosphatase
Rpe: Ribulose-phosphate 3-epimerase
RpiA: Ribose-5-phosphate isomerase
Xu5P: Xylulose-5-phosphate
Ru5P: Ribulose-5-phosphate
R5P: Ribose-5-phosphate
DHA: Dihydroxyacetone
DHAP: Dihydroxyacetone phosphate
F16dP: Fructose-1,6-bisphosphate
F6P: Fructose-6-phosphate
G3P: Glyceraldehyde-3-phosphate
E4P: Erythrose-4-phosphate
S7P: Sedoheptulose-7-phosphate
S17dP: Sedoheptulose-1,7-bisphosphate
Ile: Isoleucine
Leu: Leucine
Lys: Lysine
Met: Methionine
Phe: Phenylalanine
Thr: Threonine
Arg: Arginine
Val: Valine
His: Histidine
Asp: Aspartic acid
Ser: Serine
Glu: Glutamic acid
Gly: Glycine
Ala: Alanine
Cys: Cystine acid
Tyr: Tyrosine
Pro: Proline
