# Supplementary material for: Economical production of Pichia pastoris single cell protein from methanol at industrial pilot scale
Source: Microb Cell Fact. 2023 Sep 28;22:198. doi: 10.1186/s12934-023-02198-9 (PMC10540378; doi:10.1186/s12934-023-02198-9)
Supplement: Supplementary file 1 — Supplementary Material 1 [file 12934_2023_2198_MOESM1_ESM.docx]

**Table S2. Primers used in the RT-qPCR assay**

| **Premier** | **Sequence（from 5′ to 3′）** |
| --- | --- |
| *PAS_chr3_0841-F* | ACTTTCGGTGAGAAGGTCGT |
| *PAS_chr3_0841-R* | CCTGTTTGTTTGCTCTGGCT |
| *PAS_chr3_1028-F* | TACACTGTGGTGGCTGACAT |
| *PAS_chr3_1028-R* | ACATCCAGCACCAAACACAC |
| *PAS_chr4_0821-F* | GGTGAGGTCACTTCTCACCA |
| *PAS_chr4_0821-R* | GAGCAAGACCAGCAGACAAG |
| *PAS_chr3_0832-F* | GTCGGTATGGCCATTGGTTC |
| *PAS_chr3_0832-R* | GTCCCTCTTGCAAACAAGCA |
| *PAS_chr3_0932-F* | GCGGCCATGAATTGGTTACT |
| *PAS_chr3_0932-R* | TCCAAGTCAATGTGGTCGGA |
| *PAS_chr3_0867-F* | ACTCCCAACAATGCCTCAGA |
| *PAS_chr3_0867-R* | CGTGCTCTCCTTCGATGTTG |
| *PAS_chr3_0834-F* | ACGAGGTTGAGCAATACCCA |
| *PAS_chr3_0834-R* | GTCTGGCTTACCCTCACAGT |
| *PAS_chr3_0693-F* | GTACATGGAATGGCGTCGAG |
| *PAS_chr3_0693-R* | AGTTCAGATCGGTCCACGTT |
| *PAS_chr1-1_0319-F* | GACCTTAGCGCTTCAGTGAC |
| *PAS_chr1-1_0319-R* | GGGAGGAGAACAAAGGCTCT |
| *PAS_chr4_0152-F* | GAGGCCAGAGCTTACGAGAT |
| *PAS_chr4_0152-R* | GAGCAAGACCAGCAGTCAAG |
| *PAS_chr2-1_0771-F* | GGCCAGTGCATTAGACATGG |
| *PAS_chr2-1_0771-R* | TAACAGCAGCTGAGAACCGA |
| *PAS_chr1-4_0042-F* | CACAGCATGGATTCGCAAGA |
| *PAS_chr1-4_0042-R* | AGAGGTCACAAGCTGCTCAT |
| *PAS_chr4_0212-F* | GGGCAGCGCTTTATCAAGAA |
| *PAS_chr4_0212-R* | CGCTGTTGGCTCCTAGTTTG |
| *PAS_chr1-4_0669-F* | ACCGCTAACGGATGAAGACA |
| *PAS_chr1-4_0669-R* | AGTGCAGAGCATGAGAGGAA |
| *PAS_chr3_0277-F* | CTGACACCAACCGTCGTTAC |
| *PAS_chr3_0277-R* | ATGCCTCTTTAGCACCACCT |
| *PAS_chr2-2_0338-F* | CAGGACGAGTCTCCACTGAA |
| *PAS_chr2-2_0338-R* | ACTCCAGGGCTGCTTGTATT |
| *PAS_chr4_0815-F* | CCATCGGCTTTCGTTCTTGT |
| *PAS_chr4_0815-R* | GAGAACACGGGTGTCGAAAG |
| *PAS_chr2-1_0580-F* | ATAGATGCTGCTGAGGCCAT |
| *PAS_chr2-1_0580-R* | CGGTTCCGCCCAATATGTTT |
| *PAS_chr4_0559-F* | AGAGCTCCTCGTCTTCTTCG |
| *PAS_chr4_0559-R* | GGTTGTGGAGGAACTTGCTG |
| *PAS_chr1-1_0393-F* | CCCAAGAGTAGCTGGAGCAT |
| *PAS_chr1-1_0393-R* | GAGCAGCAACCAGTGGATTT |
| *PAS_chr2-1_0065-F* | CTCCAGAACTGTCTGCTCCA |
| *PAS_chr2-1_0065-R* | GATGAGAAGAGGCGGTGAGA |
| *ACT1-F* | AGTGTTCCCATCGGTCGTAG |
| *ACT1-R* | GGTGTGGTGCCAGATCTTTT |
