# Supplementary material for: Economical production of Pichia pastoris single cell protein from methanol at industrial pilot scale
Source: Microb Cell Fact. 2023 Sep 28;22:198. doi: 10.1186/s12934-023-02198-9 (PMC10540378; doi:10.1186/s12934-023-02198-9)
Supplement: Supplementary file 2 — Supplementary Material 2 [file 12934_2023_2198_MOESM2_ESM.docx]

**Table S1. Primers used for strain and plasmid construction in this study**

| **Premier** | **Sequence（from 5′ to 3′）** |
| --- | --- |
| *PNSII-3-up-F* | TTATAGAATTAAGTAAGTCTTTA |
| *PNSII-3-up-R* | TCGACAAGCAGAACACTAAGATCT |
| *PAOX1-F* | TTCTCTAGATCTTAGTGTTCTGCTTGTCGAGATCTAACATCCAAAGACGAAAGGT |
| *PAOX1-R* | CGTTTCGAATAATTAGTTGTTTTTTGA |
| *GDHI-F* | AGATCAAAAAACAACTAATTATTCGAAACGATGGTCCAACCACAAGAACCAGAAT |
| *GDHI-R* | CTAAAACACATCACCTTGGGCGTACAT |
| *TADH2 (GDH1)-F* | GCTATGTACGCCCAAGGTGATGTGTTTTAGGTGTATAGTCAATAATAGCCGGA |
| *TADH2 (GDH1)-R* | CTGAAAGTGAAAGCCGCTGGAA |
| *PNSII-3-down-F* | TCGGATAATTCCAGCGGCTTTCACTTTCAGAATGCTAGCCTAGTTGTCAGAG |
| *PNSII-3-down-R* | TACAACGAAGATAGAAGAATCGG |
| *gPNSII-3-1F* | CTTATACTGATGAGTCCGTGAGGACGAAACGAGTAAGCTCGTCTATAAGGCTCTTGTAGATGGGT TTTAGAGCTAGAAATAGCA |
| *gPNSII-3-1R* | ACGGGAAGTCTTTACAGTTT |
| *gPNSII-3-2F* | CTCCTAACTAAAACTGTAAAGACTTCCCGTTTAAACTTTTCTTTTCTTCT |
| *gPNSII-3-2R* | GTTTCGTCCTCACGGACTCATCAGTATAAGTTTGATTTGTTTAGGTAACT |
| *PNSI-2-up-F* | TGGTATACCGTAATTTCTCAGA |
| *PNSI-2-up-R* | TCTACGTTTTAAGATCAATCA |
| *PDAS2-F* | AAAGTGATTTGATTGATCTTAAAACGTAGAATTACTGTTTTGGGCAATCCTGT |
| *PDAS2-R* | TTTTGATGTTTGATAGTTTGATAAG |
| *GLN1-F* | TCACTCTTATCAAACTATCAAACATCAAAAATGTCTTCATCCGAAATCATTGAA |
| *GLN1-R* | TTAATCAGATTCTCTCTTGTACTC |
| *TADH2 (GLN1)-F* | ACCAAGGAGTACAAGAGAGAATCTGATTAAGTGTATAGTCAATAATAGCCGGA |
| *TADH2 (GLN1)-R* | CTGAAAGTGAAAGCCGCTGGAA |
| *PNSI-2-down-F* | TCGGATAATTCCAGCGGCTTTCACTTTCAGAGTAACAAAAAATGAAAAAATTA |
| *PNSI-2-down-R* | TTAAAGTCTTTTATTCAAATCT |
| *gPNSI-2-1F* | CCAACCCTGATGAGTCCGTGAGGACGAAACGAGTAAGCTCGTCGGTTGGTACTATGTCCAACAGTTTTAGAGCTAGAAATAGCA |
| *gPNSI-2-1R* | ACGGGAAGTCTTTACAGTTT |
| *gPNSI-2-2F* | CTCCTAACTAAAACTGTAAAGACTTCCCGTTTAAACTTTTCTTTTCTTCT |
| *gPNSI-2-2R* | GTTTCGTCCTCACGGACTCATCAGGGTTGGTTTGATTTGTTTAGGTAACT |
| *PAS_chr4_0305-up-F* | GAAGGTTCTAATACTAAGAACT |
| *PAS_chr4_0305-up-R* | AATTGGATTGAAGAGTACTAAT |
| *PAS_chr4_0305-down-F* | GCTGTACTATTAGTACTCTTCAATCCAATTAAGGGAGAAAAAGAAAAGTTAT |
| *PAS_chr4_0305-down-R* | ACAATTCAGATATTCGACGGTT |
| *gPAS_chr4_0305-1-F* | GGATGACTGATGAGTCCGTGAGGACGAAACGAGTAAGCTCGTCTCATCCTATTCTAATACATTGTTTTAGAGCTAGAAATAGCA |
| *gPAS_chr4_0305-1-R* | ACGGGAAGTCTTTACAGTTT |
| *gPAS_chr4_0305-2-F* | CTCCTAACTAAAACTGTAAAGACTTCCCGTTTAAACTTTTCTTTTCTTCT |
| *gPAS_chr4_0305-2-R* | GTTTCGTCCTCACGGACTCATCAGTCATCCTTTGATTTGTTTAGGTAACT |
