# Supplementary material for: Economical production of Pichia pastoris single cell protein from methanol at industrial pilot scale
Source: Microb Cell Fact. 2023 Sep 28;22:198. doi: 10.1186/s12934-023-02198-9 (PMC10540378; doi:10.1186/s12934-023-02198-9)
Supplement: Supplementary file 3 — Supplementary Material 3 [file 12934_2023_2198_MOESM3_ESM.docx]

**Table S4. Total enrichment results of biological pathways via KOBAS**

| **Num** | **Description** | ***P*_adjust_** | **Gene_names** |
| --- | --- | --- | --- |
| 12 | Methane metabolism | 0.000295 | *PAS_chr3_0834;PAS_chr3_0841;PAS_chr4_0821;PAS_chr3_0832;PAS_chr4_0285;PAS_chr3_0403;PAS_chr4_0152;PAS_chr3_1028;PAS_chr3_0932;PAS_chr1-1_0319;PAS_chr3_0867;PAS_chr3_0693* |
| 5 | Thiamine metabolism | 0.272749 | *PAS_chr2-1_0111;PAS_chr1-4_0141;PAS_chr3_0648;PAS_chr3_0843;PAS_chr3_0052* |
| 7 | Nucleotide excision repair | 0.707468 | *PAS_chr3_0961;PAS_chr2-1_0619;PAS_chr1-4_0556;PAS_chr4_0745;PAS_chr2-1_0195;PAS_chr2-1_0620;PAS_chr1-4_0245* |
| 3 | Tyrosine metabolism | 0.722618 | *PAS_chr4_0974;PAS_chr2-1_0307;PAS_chr3_1028* |
| 3 | Valine, leucine and isoleucine degradation | 0.722618 | *PAS_chr2-1_0107;PAS_chr1-4_0304;PAS_chr4_0248* |
| 3 | Riboflavin metabolism | 0.736739 | *PAS_chr3_1037;PAS_chr4_0081;PAS_chr4_0996* |
| 4 | Sulfur metabolism | 0.741585 | *PAS_chr2-1_0862;PAS_chr1-4_0253;PAS_chr4_0369;PAS_chr4_0330* |
| 8 | Purine metabolism | 0.741759 | *PAS_chr2-2_0219;PAS_chr2-1_0111;PAS_chr4_0613;PAS_chr1-1_0132;PAS_chr1-1_0063;PAS_chr1-4_0253;PAS_chr2-1_0771;PAS_chr3_0085* |
| 4 | Glutathione metabolism | 0.745033 | *PAS_chr3_0277;PAS_chr1-1_0029;PAS_chr2-1_0580;PAS_chr1-1_0433* |
| 2 | Nitrogen metabolism | 0.770863 | *PAS_chr2-1_0037;PAS_chr2-1_0311* |
| 14 | MAPK signaling pathway - yeast | 0.784688 | *PAS_chr3_0299;PAS_chr1-4_0533;PAS_chr2-2_0131;PAS_chr2-1_0124;PAS_chr2-1_0203;PAS_chr4_0315;PAS_chr2-1_0112;PAS_chr3_0895;PAS_chr4_0336;PAS_FragB_0058;PAS_chr2-1_0586;PAS_chr3_0303;PAS_chr4_0584;PAS_chr1-3_0061* |
| 6 | DNA replication | 0.784829 | *PAS_chr2-1_0620;PAS_chr3_0961;PAS_chr4_0279;PAS_chr2-1_0619;PAS_chr1-4_0556;PAS_chr1-1_0049* |
| 5 | Pyrimidine metabolism | 0.802525 | *PAS_chr1-1_0132;PAS_chr4_0138;PAS_chr2-1_0478;PAS_chr1-1_0163;PAS_chr1-1_0063* |
| 5 | Fructose and mannose metabolism | 0.802525 | *PAS_chr4_0754;PAS_chr1-1_0319;PAS_chr2-2_0060;PAS_chr3_0841;PAS_chr4_0339* |
| 19 | Biosynthesis of cofactors | 0.805848 | *PAS_chr1-4_0141;PAS_chr3_0648;PAS_chr4_0248;PAS_chr4_0823;PAS_chr4_0138;PAS_chr2-1_0111;PAS_chr4_0996;PAS_chr4_0081;PAS_chr3_0291;PAS_chr4_0613;PAS_chr1-3_0078;PAS_chr3_1037;PAS_chr1-4_0393;PAS_chr4_0691;PAS_chr3_1194;PAS_chr3_0052;PAS_chr3_0954;PAS_chr2-1_0871;PAS_chr3_0843* |
| 3 | ABC transporters | 0.809405 | *PAS_chr3_0858;PAS_chr3_0822;PAS_chr2-1_0613* |
| 4 | Amino sugar and nucleotide sugar metabolism | 0.818845 | *PAS_chr1-1_0393;PAS_chr4_0060;PAS_chr2-1_0065;PAS_chr2-1_0771* |
| 12 | Spliceosome | 0.820616 | *PAS_chr3_0420;PAS_chr4_0448;PAS_chr4_0309;PAS_chr2-1_0455;PAS_chr1-3_0111;PAS_chr3_1147;PAS_chr3_1041;PAS_chr1-3_0256;PAS_chr1-4_0661;PAS_chr4_0126;PAS_chr4_0538;PAS_chr1-3_0182* |
| 6 | Glycolysis / Gluconeogenesis | 0.832941 | *PAS_chr3_0403;PAS_chr1-4_0042;PAS_chr1-1_0319;PAS_chr2-1_0771;PAS_chr3_1028;PAS_chr3_0693* |
| 2 | Cyanoamino acid metabolism | 0.836668 | *PAS_chr2-2_0481;PAS_chr2-1_0037* |
| 4 | Propanoate metabolism | 0.858036 | *PAS_chr2-1_0107;PAS_chr1-4_0304;PAS_chr4_0336;PAS_chr3_0403* |
| 6 | Pyruvate metabolism | 0.863536 | *PAS_chr4_0272;PAS_chr3_0403;PAS_chr1-4_0304;PAS_chr4_0336;PAS_chr4_0815;PAS_chr3_1028* |
| 2 | Butanoate metabolism | 0.870274 | *PAS_chr2-1_0107;PAS_chr1-4_0304* |
| 1 | Lipoic acid metabolism | 0.880404 | *PAS_chr4_0691* |
| 4 | Arginine biosynthesis | 0.924496 | *PAS_chr4_0974;PAS_chr1-1_0127;PAS_chr3_0623;PAS_chr2-1_0311* |
| 4 | Tryptophan metabolism | 0.924496 | *PAS_chr3_1194;PAS_chr1-4_0304;PAS_chr2-2_0131;PAS_chr2-1_0037* |
| 7 | Basal transcription factors | 0.925415 | *PAS_chr2-1_0838;PAS_chr1-4_0265;PAS_FragD_0016;PAS_chr4_0745;PAS_chr2-2_0231;PAS_chr1-4_0012;PAS_chr1-4_0245* |
| 2 | Sulfur relay system | 0.927271 | *PAS_chr1-4_0547;PAS_chr1-3_0292* |
| 3 | Mismatch repair | 0.935506 | *PAS_chr1-4_0556;PAS_chr3_0961;PAS_chr2-1_0619* |
| 3 | beta-Alanine metabolism | 0.938103 | *PAS_chr2-1_0107;PAS_chr2-1_0307;PAS_chr4_0823* |
| 5 | Glyoxylate and dicarboxylate metabolism | 0.948235 | *PAS_chr4_0815;PAS_chr1-4_0304;PAS_chr2-2_0131;PAS_chr3_0932;PAS_chr3_0403* |
| 1 | Monobactam biosynthesis | 0.948729 | *PAS_chr1-4_0253* |
| 6 | Pentose phosphate pathway | 0.954875 | *PAS_chr3_0277;PAS_chr2-2_0338;PAS_chr2-1_0771;PAS_chr4_0212;PAS_chr1-1_0319;PAS_chr1-4_0669* |
| 1 | Taurine and hypotaurine metabolism | 0.956197 | *PAS_chr2-1_0311* |
| 2 | Phenylalanine metabolism | 0.958508 | *PAS_chr4_0974;PAS_chr2-1_0307* |
| 14 | Ribosome | 0.962198 | *PAS_chr4_0412;PAS_chr1-4_0239;PAS_c131_0014;PAS_chr3_1200;PAS_chr2-1_0728;PAS_chr1-3_0115;PAS_chr1-1_0076;PAS_chr1-1_0183;PAS_chr1-1_0382;PAS_chr2-1_0482;PAS_chr2-1_0481;PAS_chr4_0107;PAS_chr1-4_0504;PAS_chr1-1_0219* |
| 6 | Peroxisome | 0.971607 | *PAS_chr2-2_0131;PAS_chr3_0822;PAS_chr2-1_0504;PAS_chr2-1_0580;PAS_chr3_0099;PAS_chr2-1_0230* |
| 7 | Cysteine and methionine metabolism | 0.986165 | *PAS_chr2-1_0422;PAS_chr4_0280;PAS_chr4_0815;PAS_chr4_0248;PAS_chr4_0330;PAS_chr4_0974;PAS_chr2-1_0862* |
| 2 | Fatty acid degradation | 0.996773 | *PAS_chr1-4_0304;PAS_chr3_1028* |
| 2 | Oxidative phosphorylation | 0.999157 | *PAS_chr3_0704;PAS_chr4_0892* |
| 6 | Alanine, aspartate and glutamate metabolism | 1 | *PAS_chr2-2_0481;PAS_chr2-1_0107;PAS_chr4_0613;PAS_chr4_0138;PAS_chr4_0974;PAS_chr2-1_0311* |
| 3 | Pantothenate and CoA biosynthesis | 1 | *PAS_chr4_0248;PAS_chr4_0823;PAS_chr2-1_0871* |
| 3 | Homologous recombination | 1 | *PAS_FragB_0039;PAS_chr3_0961;PAS_chr3_0904* |
| 2 | Base excision repair | 1 | *PAS_chr2-1_0620;PAS_chr2-2_0280* |
| 1 | Other types of O-glycan biosynthesis | 1 | *PAS_chr1-3_0138* |
| 1 | Fatty acid elongation | 1 | *PAS_chr3_0602* |
| 2 | Porphyrin and chlorophyll metabolism | 1 | *PAS_chr1-3_0078;PAS_chr3_0954* |
| 3 | Glycerolipid metabolism | 1 | *PAS_chr3_0512;PAS_chr3_0841;PAS_chr3_0986* |
| 2 | Terpenoid backbone biosynthesis | 1 | *PAS_chr1-4_0304;PAS_chr1-4_0314* |
| 1 | Selenocompound metabolism | 1 | *PAS_chr1-4_0253* |
| 1 | Ubiquinone and other terpenoid-quinone biosynthesis | 1 | *PAS_chr3_0291* |
| 3 | RNA polymerase | 1 | *PAS_chr2-2_0309;PAS_chr2-2_0083;PAS_chr2-1_0554* |
| 1 | Vitamin B6 metabolism | 1 | *PAS_chr1-4_0393* |
| 1 | Hippo signaling pathway - multiple species | 1 | *PAS_chr3_0727* |
| 2 | Phenylalanine, tyrosine and tryptophan biosynthesis | 1 | *PAS_chr4_0974;PAS_chr4_0050* |
| 3 | Longevity regulating pathway - multiple species | 1 | *PAS_chr2-1_0230;PAS_chr2-2_0131;PAS_chr3_0893* |
| 1 | Ascorbate and aldarate metabolism | 1 | *PAS_chr2-1_0197* |
| 1 | Galactose metabolism | 1 | *PAS_chr2-1_0771* |
| 1 | Valine, leucine and isoleucine biosynthesis | 1 | *PAS_chr4_0248* |
| 10 | Cell cycle - yeast | 1 | *PAS_chr2-1_0508;PAS_chr3_0395;PAS_chr1-4_0054;PAS_chr2-2_0153;PAS_chr3_0599;PAS_chr4_0526;PAS_chr2-2_0307;PAS_chr3_1239;PAS_FragD_0025;PAS_chr2-2_0298* |
| 1 | Biosynthesis of unsaturated fatty acids | 1 | *PAS_chr3_0602* |
| 3 | Glycine, serine and threonine metabolism | 1 | *PAS_chr4_0285;PAS_chr2-1_0307;PAS_chr3_0693* |
| 3 | Phagosome | 1 | *PAS_chr2-1_0186;PAS_chr4_0623;PAS_chr3_0704* |
| 2 | Citrate cycle (TCA cycle) | 1 | *PAS_chr2-1_0580;PAS_chr4_0815* |
| 3 | Mitophagy - yeast | 1 | *PAS_chr4_0315;PAS_chr3_0895;PAS_chr2-1_0124* |
| 3 | Glycerophospholipid metabolism | 1 | *PAS_chr3_0845;PAS_chr3_0512;PAS_chr3_1180* |
| 2 | Arginine and proline metabolism | 1 | *PAS_chr4_0974;PAS_chr4_0823* |
| 1 | Histidine metabolism | 1 | *PAS_chr1-4_0234* |
| 7 | Meiosis - yeast | 1 | *PAS_chr3_0395;PAS_chr1-4_0054;PAS_chr3_0599;PAS_chr4_0526;PAS_chr1-3_0041;PAS_chr3_1239;PAS_chr4_0878* |
| 1 | Nicotinate and nicotinamide metabolism | 1 | *PAS_chr1-1_0132* |
| 1 | Steroid biosynthesis | 1 | *PAS_chr4_0198* |
| 4 | RNA degradation | 1 | *PAS_chr1-4_0283;PAS_chr1-3_0256;PAS_chr3_0581;PAS_chr4_0448* |
| 1 | Lysine degradation | 1 | *PAS_chr1-4_0304* |
| 3 | mRNA surveillance pathway | 1 | *PAS_chr1-4_0283;PAS_chr2-1_0528;PAS_chr4_0800* |
| 1 | Phosphatidylinositol signaling system | 1 | *PAS_chr2-1_0124* |
| 1 | Inositol phosphate metabolism | 1 | *PAS_chr2-2_0113* |
| 4 | Nucleocytoplasmic transport | 1 | *PAS_chr2-1_0562;PAS_chr2-1_0528;PAS_chr4_0800;PAS_chr3_0420* |
| 1 | Glycosylphosphatidylinositol (GPI)-anchor biosynthesis | 1 | *PAS_chr1-4_0070* |
| 5 | Autophagy - yeast | 1 | *PAS_chr4_0062;PAS_chr3_0895;PAS_chr1-4_0548;PAS_chr1-4_0606;PAS_chr3_0893* |
| 5 | Protein processing in endoplasmic reticulum | 1 | *PAS_chr3_0538;PAS_chr4_0991;PAS_chr1-4_0231;PAS_chr3_1104;PAS_chr3_0249* |
| 1 | Starch and sucrose metabolism | 1 | *PAS_chr2-1_0771* |
| 1 | Various types of N-glycan biosynthesis | 1 | *PAS_chr3_0787* |
| 4 | Ribosome biogenesis in eukaryotes | 1 | *PAS_chr1-1_0335;PAS_chr3_1041;PAS_chr4_0747;PAS_chr1-4_0015* |
| 1 | N-Glycan biosynthesis | 1 | *PAS_chr1-4_0070* |
| 2 | Ubiquitin mediated proteolysis | 1 | *PAS_chr2-1_0119;PAS_chr1-1_0363* |
| 1 | Aminoacyl-tRNA biosynthesis | 1 | *PAS_chr2-1_0123* |
| 3 | Endocytosis | 1 | *PAS_chr2-1_0112;PAS_chr3_1047;PAS_chr2-1_0389* |
