# Supplementary material for: Economical production of Pichia pastoris single cell protein from methanol at industrial pilot scale
Source: Microb Cell Fact. 2023 Sep 28;22:198. doi: 10.1186/s12934-023-02198-9 (PMC10540378; doi:10.1186/s12934-023-02198-9)
Supplement: Supplementary file 4 — Supplementary Material 4 [file 12934_2023_2198_MOESM4_ESM.docx]

**Table S3. DEGs identified by RNA-seq**

| **Gene name** | **Gene description** | **Log_2_FC(HTX_33/WT_30)** | ***P*_adjust_** | **Regulate** |
| --- | --- | --- | --- | --- |
| *PAS_chr1-1_0030* | General amino acid permease | 2.966749 | 0 | up |
| *PAS_chr3_0030* | Hypothetical protein | -3.69439 | 1.41E-302 | down |
| *PAS_chr1-4_0479* | General amino acid permease | 2.739679 | 4.78E-227 | up |
| *PAS_FragB_0067* | Mucin-like protein | 3.070665 | 5.67E-210 | up |
| *PAS_chr3_0066* | Peptide methionine sulfoxide reductase, reverses the oxidation of methionine residues | -2.73737 | 4.92E-199 | down |
| *PAS_chr2-1_0659* | Proline permease, required for high-affinity transport of proline | 2.830963 | 6.42E-191 | up |
| *PAS_chr4_0152* | Hypothetical protein | -2.38118 | 1.59E-180 | down |
| *PAS_chr2-1_0383* | L-homoserine-O-acetyltransferase, catalyzes the conversion of homoserine to O-acetyl homoserine | -2.32628 | 1.89E-162 | down |
| *PAS_chr2-2_0482* | hypothetical protein | 3.597591 | 3.21E-161 | up |
| *PAS_chr4_0047* | Peripheral membrane protein located at Vid (vacuole import and degradation) vesicles | -2.56101 | 4.90E-157 | down |
| *PAS_chr2-1_0504* | Peroxisomal membrane protein | -1.86571 | 2.53E-130 | down |
| *PAS_chr4_0330* | Methionine and cysteine synthase (O-acetyl homoserine-O-acetyl serine sulfhydrylase) | -2.21389 | 3.66E-123 | down |
| *PAS_chr3_1071* | Translation elongation factor EF-1 gamma | -1.57046 | 9.93E-110 | down |
| *PAS_chr4_0287* | Dicarboxylic amino acid permease | 1.645895 | 6.69E-103 | up |
| *PAS_chr2-1_0311* | NAD(+)-dependent glutamate dehydrogenase, degrades glutamate to ammonia and alpha-ketoglutarate | 1.70763 | 1.78E-99 | up |
| *PAS_chr1-4_0042* | Glucose-6-phosphate 1-epimerase (hexose-6-phosphate mutarotase) | -1.46188 | 5.85E-97 | down |
| *PAS_chr2-1_0017* | Heme-dependent repressor of hypoxic genes | 1.858171 | 4.32E-95 | up |
| *PAS_chr2-2_0147* | Hypothetical protein | -2.08912 | 5.53E-93 | down |
| *PAS_chr4_0157* | Histone variant H2AZ, exchanged for histone H2A in nucleosomes by the SWR1 complex | -2.36943 | 3.99E-92 | down |
| *PAS_chr1-4_0516* | Putative transcription factor | -1.84385 | 1.33E-91 | down |
| *PAS_chr4_0815* | Mitochondrial malate dehydrogenase, catalyzes interconversion of malate and oxaloacetate | -1.8312 | 5.09E-91 | down |
| *PAS_chr1-4_0253* | ATP sulfurylase, catalyzes the primary step of intracellular sulfate activation | -1.96741 | 7.03E-86 | down |
| *PAS_chr1-1_0319* | Fructose 1,6-bisphosphate aldolase, required for glycolysis and gluconeogenesis | -1.47341 | 1.23E-84 | down |
| *PAS_chr2-1_0009* | Hypothetical protein | 2.359253 | 3.43E-82 | up |
| *PAS_chr1-4_0531* | Hypothetical protein | -1.79016 | 4.34E-81 | down |
| *PAS_chr1-3_0043* | Pheromone-regulated protein, predicted to have 5 transmembrane segments | 1.993202 | 3.45E-80 | up |
| *PAS_chr1-1_0433* | Mitochondrial peroxiredoxin (1-Cys Prx) with thioredoxin peroxidase activity | -1.78133 | 6.77E-80 | down |
| *PAS_chr4_0559* | Putative chitin transglycosidase, cell wall protein | 1.621509 | 2.61E-79 | up |
| *PAS_chr2-2_0338* | Transaldolase, enzyme in the non-oxidative pentose phosphate pathway | -1.51174 | 5.25E-78 | down |
| *PAS_chr1-1_0417* | Putative transmembrane protein involved in export of ammonia | -1.61704 | 6.92E-77 | down |
| *PAS_chr3_0015* | Hypothetical protein | -1.87709 | 2.68E-76 | down |
| *PAS_chr3_0374* | Hypothetical protein | -1.54086 | 7.39E-76 | down |
| *PAS_chr1-1_0341* | Lysine permease | 1.949736 | 2.28E-72 | up |
| *PAS_chr1-1_0393* | Chitin synthase I | 1.764487 | 1.58E-71 | up |
| *PAS_chr2-2_0177* | Putative protein of unknown function | -1.45818 | 2.26E-71 | down |
| *PAS_chr3_0337* | Hypothetical protein | 1.880899 | 2.81E-71 | up |
| *PAS_chr1-4_0601* | Plasma membrane arginine permease, requires phosphatidyl ethanolamine (PE) for localization | 1.433304 | 4.54E-71 | up |
| *PAS_chr3_0076* | Protein of unknown function, has similarity to Pry1p and Pry3p and to the plant PR-1 class of pathog | -1.79047 | 1.44E-66 | down |
| *PAS_chr3_0040* | Mitochondrial inner membrane transporter, exports 2-oxoadipate and 2-oxoglutarate from the mitochond | -1.6823 | 2.18E-65 | down |
| *PAS_chr3_0648* | Thiazole synthase, catalyzes formation of the thiazole moiety of thiamin pyrophosphate | -1.32396 | 3.99E-65 | down |
| *PAS_chr2-1_0701* | Hypothetical protein | -1.47162 | 3.00E-64 | down |
| *PAS_chr2-1_0428* | One of two nearly identical (see HTB1) histone H2B subtypes | -1.40752 | 8.71E-64 | down |
| *PAS_chr4_0922* | hypothetical protein | -1.4712 | 1.90E-63 | down |
| *PAS_chr4_0342* | Acid trehalase required for utilization of extracellular trehalose | 1.383694 | 2.64E-63 | up |
| *PAS_chr1-1_0085* | Hypothetical protein | -1.84854 | 2.82E-63 | down |
| *PAS_chr1-4_0229* | Hypothetical protein | -2.17271 | 3.42E-62 | down |
| *PAS_chr3_0125* | Putative protein of unknown function | -1.64463 | 4.63E-62 | down |
| *PAS_chr2-1_0489* | Myo-inositol transporter with strong similarity to the major myo-inositol transporter Itr1p | -1.39906 | 1.06E-60 | down |
| *PAS_chr4_0358* | Hypothetical protein | 1.626854 | 1.19E-60 | up |
| *PAS_chr1-4_0699* | hypothetical protein | -1.19659 | 1.26E-59 | down |
| *PAS_chr1-4_0547* | peroxiredoxin | -1.313 | 3.99E-59 | down |
| *PAS_chr2-2_0489* | hypothetical protein | -1.80291 | 7.92E-59 | down |
| *PAS_chr4_0498* | Suppressor of sphingoid long chain base (LCB) sensitivity of an LCB-lyase mutation | -1.48646 | 1.88E-57 | down |
| *PAS_chr1-4_0394* | Ammonium permease involved in regulation of pseudohyphal growth | -2.04957 | 3.11E-57 | down |
| *PAS_chr2-1_0707* | Probable serine protease of the SPS plasma membrane amino acid sensor system (Ssy1p-Ptr3p-Ssy5p), wh | 1.927592 | 1.01E-56 | up |
| *PAS_chr3_0895* | Serine/threonine MAP kinase | 1.870124 | 1.13E-56 | up |
| *PAS_chr2-2_0226* | Hypothetical protein | 1.555534 | 1.40E-56 | up |
| *PAS_chr4_0784* | Putative channel-like protein | -1.38122 | 1.95E-56 | down |
| *PAS_chr4_0948* | hypothetical protein | -1.88422 | 9.80E-56 | down |
| *PAS_chr4_0212* | Ribose-5-phosphate ketol-isomerase | -1.51424 | 2.12E-55 | down |
| *PAS_chr3_0811* | Hypothetical protein | -1.49307 | 6.38E-55 | down |
| *PAS_chr3_0099* | Mitochondrial NAD+ transporter, involved in the transport of NAD+ into the mitochondria (see also YE | -1.39952 | 1.07E-54 | down |
| *PAS_chr2-1_0422* | Acireductone dioxygenease involved in the methionine salvage pathway | -1.3054 | 2.30E-52 | down |
| *PAS_chr3_0299* | Aspartic protease, attached to the plasma membrane via a glycosylphosphatidylinositol (GPI) anchor | -1.48173 | 2.55E-52 | down |
| *PAS_chr3_0867* | Non-essential intracellular esterase that can function as an S-formylglutathione hydrolase | -1.48556 | 2.88E-52 | down |
| *PAS_chr2-1_0203* | Phosphotyrosine-specific protein phosphatase involved in the inactivation of mitogen-activated prote | 1.338421 | 1.53E-51 | up |
| *PAS_chr1-1_0159* | Protein of unknown function, required for normal localization of actin patches | -1.852 | 4.28E-51 | down |
| *PAS_chr2-1_0866* | hypothetical protein | 1.926552 | 5.16E-51 | up |
| *PAS_chr4_0588* | Hypothetical protein | -1.56053 | 6.75E-51 | down |
| *PAS_chr4_0754* | Peroxisomal 2,4-dienoyl-CoA reductase, auxiliary enzyme of fatty acid beta-oxidation | -1.30166 | 1.00E-50 | down |
| *PAS_chr2-2_0199* | One of two identical histone H3 proteins (see also HHT2) | -1.3493 | 1.62E-50 | down |
| *PAS_chr2-1_0365* | Putative protein of unknown function | -1.70383 | 2.69E-50 | down |
| *PAS_chr2-2_0214* | Protein of unknown function involved in rRNA and ribosome biosynthesis | 1.569315 | 7.55E-50 | up |
| *PAS_chr4_0823* | Polyamine oxidase, converts spermine to spermidine | -1.61824 | 2.17E-49 | down |
| *PAS_chr4_0496* | Peroxisomal ubiquitin conjugating enzyme | -1.40244 | 4.64E-49 | down |
| *PAS_chr4_0439* | Plasma membrane transporter for both urea and polyamines | -2.20221 | 5.26E-49 | down |
| *PAS_chr4_0369* | Subunit alpha of assimilatory sulfite reductase | -1.4186 | 5.29E-49 | down |
| *PAS_chr2-1_0429* | One of two nearly identical (see also HTA1) histone H2A subtypes | -1.14742 | 3.51E-48 | down |
| *PAS_chr3_0771* | Hypothetical protein | -1.27802 | 3.51E-48 | down |
| *PAS_chr2-1_0404* | Transcriptional repressor and activator | 1.5528 | 7.64E-48 | up |
| *PAS_chr3_0932* | NAD(+)-dependent formate dehydrogenase, may protect cells from exogenous formate | -1.12982 | 2.70E-47 | down |
| *PAS_chr3_0697* | Hypothetical protein | -1.73215 | 7.93E-47 | down |
| *PAS_chr2-1_0301* | Voltage-gated high-affinity calcium channel | 1.869105 | 2.20E-46 | up |
| *PAS_chr4_0346* | Catalytic subunit of the NatB N-terminal acetyltransferase | -1.60953 | 3.16E-46 | down |
| *PAS_chr2-1_0862* | hypothetical protein | -1.31207 | 7.25E-46 | down |
| *PAS_chr3_1028* | S-(hydroxymethyl)glutathione dehydrogenase | -1.20301 | 8.17E-46 | down |
| *PAS_chr2-2_0064* | Protein of the SUN family (Sim1p, Uth1p, Nca3p, Sun4p) that may participate in DNA replication | -1.25368 | 8.89E-46 | down |
| *PAS_chr2-2_0208* | Hypothetical protein | 1.537551 | 2.06E-45 | up |
| *PAS_chr1-4_0164* | Protein of unknown function, has similarity to Pry1p and Pry3p | -1.46375 | 4.28E-45 | down |
| *PAS_chr2-1_0479* | Pleckstrin homology domain containing protein proposed to function as a glycerol channel activator | 1.600095 | 6.48E-45 | up |
| *PAS_chr3_1037* | Lumazine synthase (6,7-dimethyl-8-ribityllumazine synthase, also known as DMRL synthase) | -1.37295 | 7.58E-45 | down |
| *PAS_chr1-4_0703* | hypothetical protein | -1.37 | 1.67E-44 | down |
| *PAS_chr1-1_0063* | Apyrase with wide substrate specificity | 1.866544 | 6.56E-44 | up |
| *PAS_chr4_0974* | hypothetical protein | -1.21854 | 8.16E-44 | down |
| *PAS_chr4_0363* | Hypothetical protein | 1.135915 | 1.80E-43 | up |
| *PAS_chr3_0414* | Calcineurin A | 1.205206 | 1.96E-43 | up |
| *PAS_chr3_0858* | Plasma membrane ATP-binding cassette (ABC) transporter required for the export of a-factor | -1.94879 | 3.05E-43 | down |
| *PAS_chr1-4_0304* | Acetyl-CoA C-acetyltransferase (acetoacetyl-CoA thiolase), cytosolic enzyme | -1.17867 | 4.58E-43 | down |
| *PAS_chr3_0303* | Aspartic protease, attached to the plasma membrane via a glycosylphosphatidylinositol (GPI) anchor | -1.19321 | 5.55E-43 | down |
| *PAS_chr1-1_0479* | hypothetical protein | -1.28289 | 2.31E-42 | down |
| *PAS_chr1-3_0032* | EH domain-containing protein | 1.539962 | 2.37E-42 | up |
| *PAS_chr2-2_0236* | Hypothetical protein | -1.21952 | 2.50E-42 | down |
| *PAS_chr2-1_0230* | Mitochondrial ribosomal protein of the small subunit | -1.39776 | 3.78E-42 | down |
| *PAS_chr2-1_0571* | Putative protein of unknown function | -1.18853 | 5.13E-42 | down |
| *PAS_chr1-4_0361* | Hypothetical protein | -1.90335 | 5.48E-42 | down |
| *PAS_chr3_0208* | Hypothetical protein | -1.5946 | 7.55E-42 | down |
| *PAS_chr4_0241* | Putative ATPase of the AAA family | 1.366857 | 1.56E-41 | up |
| *PAS_chr3_0336* | Hypothetical protein | -1.17163 | 2.39E-41 | down |
| *PAS_chr3_0581* | 3'-5' exoribonuclease involved in rRNA processing | -1.48828 | 2.60E-41 | down |
| *PAS_chr3_0789* | Ribose methyltransferase | -1.48587 | 3.57E-41 | down |
| *PAS_chr1-4_0314* | Farnesyl pyrophosphate synthetase | -1.24849 | 3.63E-41 | down |
| *PAS_chr4_0821* | Alcohol oxidase | -1.11703 | 7.78E-41 | down |
| *PAS_chr1-4_0054* | G1 cyclin involved in cell cycle progression | 1.489278 | 7.87E-41 | up |
| *PAS_chr3_0002* | Hypothetical protein | 1.467351 | 8.49E-41 | up |
| *PAS_chr3_0085* | Phosphoribosylaminoimidazole carboxylase, catalyzes a step in the 'de novo' purine nucleotide biosyn | -1.22593 | 9.47E-41 | down |
| *PAS_chr3_0904* | Strand exchange protein, forms a helical filament with DNA that searches for homology | -1.60517 | 1.35E-40 | down |
| *PAS_chr3_1230* | ATG30 | -1.33875 | 2.16E-40 | down |
| *PAS_chr2-1_0854* | hypothetical protein | -2.04605 | 5.15E-40 | down |
| *PAS_chr2-1_0807* | hypothetical protein | 1.677465 | 1.14E-39 | up |
| *PAS_chr3_1020* | Hypothetical protein | -1.11652 | 1.14E-39 | down |
| *PAS_chr2-2_0219* | Guanylate kinase, converts GMP to GDP | -1.48536 | 1.51E-39 | down |
| *PAS_chr3_0893* | Protein kinase involved in transcriptional activation of osmostress-responsive genes | 1.446682 | 2.95E-39 | up |
| *PAS_chr3_0764* | Beta subunit of Type II geranylgeranyltransferase | -1.22338 | 8.58E-39 | down |
| *PAS_chr1-3_0061* | Phosphotyrosine-specific protein phosphatase | 1.223089 | 1.87E-38 | up |
| *PAS_chr4_0412* | 60S acidic ribosomal protein P1 | -1.4344 | 3.29E-38 | down |
| *PAS_chr4_0623* | Alpha-tubulin | -1.28471 | 4.69E-38 | down |
| *PAS_chr3_1200* | 40S ribosomal protein S12 | -1.54508 | 5.28E-38 | down |
| *PAS_chr2-1_0481* | 40S ribosomal protein S14 | -1.49318 | 5.36E-38 | down |
| *PAS_chr1-1_0163* | Deoxycytidine monophosphate (dCMP) deaminase required for dCTP and dTTP synthesis | -1.59301 | 6.11E-38 | down |
| *PAS_chr3_0841* | Dihydroxyacetone kinase, required for detoxification of dihydroxyacetone (DHA) | -1.02452 | 6.45E-38 | down |
| *PAS_FragB_0039* | DNA-dependent ATPase | -1.1711 | 3.39E-37 | down |
| *PAS_chr1-3_0115* | 40S ribosomal protein S3 | -1.07045 | 3.67E-37 | down |
| *PAS_chr1-1_0382* | 60S ribosomal protein L9 | -1.09759 | 3.79E-37 | down |
| *PAS_chr4_0336* | Putative dihydrokaempferol 4-reductase | -1.26648 | 4.69E-37 | down |
| *PAS_chr2-2_0200* | One of two identical histone H4 proteins (see also HHF2) | -1.12793 | 4.93E-37 | down |
| *PAS_chr1-4_0393* | Member of a stationary phase-induced gene family | 1.224627 | 7.43E-37 | up |
| *PAS_chr4_0540* | Zinc cluster transcriptional activator | 1.341409 | 1.15E-36 | up |
| *PAS_chr3_0832* | Transketolase, similar to Tkl2p | -1.08356 | 1.47E-36 | down |
| *PAS_chr2-1_0542* | Putative benzil reductase | -1.3561 | 4.01E-36 | down |
| *PAS_chr1-3_0138* | Alpha-1,2-mannosyltransferase involved in O-and N-linked protein glycosylation | 1.418917 | 8.36E-36 | up |
| *PAS_chr3_0504* | Hypothetical protein | 1.241239 | 2.60E-35 | up |
| *PAS_chr1-4_0548* | Vacuolar proteinase B (yscB), a serine protease of the subtilisin family | -1.20885 | 5.43E-35 | down |
| *PAS_chr1-3_0201* | Hypothetical protein | -1.44353 | 5.50E-35 | down |
| *PAS_chr4_0562* | Similar to globins and has a functional heme-binding domain | -1.44354 | 1.47E-34 | down |
| *PAS_chr3_0834* | Transketolase, similar to Tkl2p | -1.03847 | 1.56E-34 | down |
| *PAS_chr2-2_0131* | Catalase A, breaks down hydrogen peroxide in the peroxisomal matrix formed by acyl-CoA oxidase (Pox1 | -1.02928 | 1.83E-34 | down |
| *PAS_chr1-1_0146* | Putative protein of unknown function | -1.40266 | 1.91E-34 | down |
| *PAS_chr2-1_0577* | Methyltransferase required for synthesis of diphthamide | -1.35393 | 2.22E-34 | down |
| *PAS_chr1-1_0155* | Nuclear thiol peroxidase, functions as an alkyl-hydroperoxide reductase during post-diauxic growth | -1.40152 | 4.08E-34 | down |
| *PAS_chr1-4_0090* | Polyamine transport protein specific for spermine | -1.0283 | 6.33E-34 | down |
| *PAS_chr1-1_0013* | Hypothetical protein | 1.163015 | 1.22E-33 | up |
| *PAS_chr2-2_0324* | Hypothetical protein | 1.437161 | 1.62E-33 | up |
| *PAS_chr1-4_0420* | Mitochondrial protein, putative inner membrane transporter | -1.44022 | 2.20E-33 | down |
| *PAS_chr4_0285* | Phosphoserine phosphatase of the phosphoglycerate pathway, involved in serine and glycine biosynthes | -1.39594 | 2.59E-33 | down |
| *PAS_chr4_0272* | Cytochrome b2 (L-lactate cytochrome-c oxidoreductase) | -1.1185 | 3.06E-33 | down |
| *PAS_chr4_0248* | Mitochondrial branched-chain amino acid aminotransferase, homolog of murine ECA39 | -1.16743 | 3.40E-33 | down |
| *PAS_chr4_0678* | Putative protein of unknown function | 1.164698 | 5.65E-33 | up |
| *PAS_chr4_0914* | hypothetical protein | 1.713751 | 9.12E-33 | up |
| *PAS_c131_0003* | Hypothetical protein | -1.11913 | 9.44E-33 | down |
| *PAS_chr4_0107* | 60S ribosomal protein L2 | -1.06237 | 2.94E-32 | down |
| *PAS_chr2-1_0210* | Hypothetical protein | 1.018957 | 3.23E-32 | up |
| *PAS_chr1-1_0183* | 60S ribosomal protein L1 | -1.27637 | 3.67E-32 | down |
| *PAS_chr2-1_0186* | Alpha-tubulin | -1.02989 | 4.15E-32 | down |
| *PAS_chr2-1_0021* | Hypothetical protein | -1.40221 | 4.28E-32 | down |
| *PAS_chr3_0105* | Hypothetical protein | -1.2765 | 4.60E-32 | down |
| *PAS_FragB_0007* | Protein VTS1 | 1.178088 | 9.20E-32 | up |
| *PAS_chr3_1231* | hypothetical protein | -1.2831 | 9.23E-32 | down |
| *PAS_chr2-1_0871* | hypothetical protein | 1.011592 | 9.67E-32 | up |
| *PAS_chr4_0877* | Mitochondrial dicarboxylate carrier, integral membrane protein | -1.35793 | 9.86E-32 | down |
| *PAS_chr1-4_0312* | Putative protein of unknown function | 1.671679 | 1.02E-31 | up |
| *PAS_chr4_0280* | Putative methylthio-ribulose-1-phosphate dehydratase | -1.43866 | 1.13E-31 | down |
| *PAS_chr1-4_0537* | Protein of unknown function, similar to Listeria monocytogenes major sigma factor | -1.38428 | 1.20E-31 | down |
| *PAS_chr3_0028* | Putative protein with similarity to the allantoate permease (Dal5p) subfamily of the major facilitat | -1.51806 | 1.35E-31 | down |
| *PAS_chr2-1_0111* | Mitochondrial adenylate kinase, catalyzes the reversible synthesis of GTP and AMP from GDP and ADP | -1.39359 | 1.60E-31 | down |
| *PAS_chr2-1_0810* | hypothetical protein | -1.1426 | 1.74E-31 | down |
| *PAS_chr2-1_0505* | Subunit of the GINS complex (Sld5p, Psf1p, Psf2p, Psf3p) | -1.31476 | 2.25E-31 | down |
| *PAS_chr2-2_0034* | Alkaline phosphatase specific for p-nitrophenyl phosphate | -1.25754 | 2.54E-31 | down |
| *PAS_chr4_0050* | Chorismate mutase, catalyzes the conversion of chorismate to prephenate | -1.01199 | 2.73E-31 | down |
| *PAS_chr4_0181* | High-affinity copper transporter of the plasma membrane | -1.0636 | 3.50E-31 | down |
| *PAS_chr4_0081* | Riboflavin synthase | -1.03224 | 6.12E-31 | down |
| *PAS_chr2-1_0197* | D-Arabinono-1,4-lactone oxidase, catalyzes the final step in biosynthesis of D-erythroascorbic acid | -1.57561 | 6.33E-31 | down |
| *PAS_chr1-3_0301* | hypothetical protein | -1.53504 | 8.88E-31 | down |
| *PAS_chr3_0704* | Subunit E of the eight-subunit V1 peripheral membrane domain of the vacuolar H+-ATPase (V-ATPase) | -1.00324 | 1.22E-30 | down |
| *PAS_chr2-1_0580* | Cytosolic NADP-specific isocitrate dehydrogenase | -1.29622 | 1.43E-30 | down |
| *PAS_chr1-4_0239* | 60S ribosomal protein L34 | -1.1641 | 1.49E-30 | down |
| *PAS_chr1-1_0029* | Putative protein of unknown function | -1.36545 | 1.68E-30 | down |
| *PAS_chr3_0822* | Subunit of a heterodimeric peroxisomal ATP-binding cassette transporter complex (Pxa1p-Pxa2p) | 1.153117 | 1.90E-30 | up |
| *PAS_chr3_1226* | hypothetical protein | 1.214728 | 1.94E-30 | up |
| *PAS_chr1-3_0256* | Hypothetical protein | -1.66958 | 4.90E-30 | down |
| *PAS_chr1-1_0049* | Ribonuclease H2 catalytic subunit, removes RNA primers during Okazaki fragment synthesis | -1.16072 | 5.60E-30 | down |
| *PAS_chr1-4_0265* | Hypothetical protein | -1.05849 | 5.69E-30 | down |
| *PAS_chr1-4_0155* | Hypothetical protein | 1.282729 | 6.76E-30 | up |
| *PAS_chr3_0249* | Hypothetical protein | -1.18756 | 6.76E-30 | down |
| *PAS_chr1-4_0282* | Histone H1, a linker histone required for nucleosome packaging at restricted sites | -1.10511 | 7.66E-30 | down |
| *PAS_chr2-2_0488* | hypothetical protein | -1.00134 | 8.05E-30 | down |
| *PAS_chr4_0044* | Hypothetical protein | -1.18103 | 8.52E-30 | down |
| *PAS_chr2-1_0664* | Hypothetical protein | 1.184898 | 8.85E-30 | up |
| *PAS_chr1-4_0231* | Essential phosphoprotein component (p150) of the COPII coat of secretory pathway vesicles | 1.382748 | 9.45E-30 | up |
| *PAS_chr4_0613* | Adenylosuccinate synthase | -1.21408 | 1.04E-29 | down |
| *PAS_chr2-2_0022* | Ubiquitin-binding component of the Rsp5p E3-ubiquitin ligase complex, functional homolog of Bul2p | 1.263691 | 2.20E-29 | up |
| *PAS_chr3_0016* | Hypothetical protein | -1.50546 | 2.26E-29 | down |
| *PAS_chr1-4_0660* | hypothetical protein | 1.142198 | 3.51E-29 | up |
| *PAS_chr4_0681* | GTPase-activating protein (RhoGAP) for Rho3p and Rho4p | 1.089624 | 3.51E-29 | up |
| *PAS_chr3_0787* | Alpha-1,2-mannosyltransferase | 1.01001 | 5.43E-29 | up |
| *PAS_chr4_0630* | Hypothetical protein | -1.64242 | 5.82E-29 | down |
| *PAS_chr2-2_0460* | hypothetical protein | -1.75029 | 5.94E-29 | down |
| *PAS_chr1-1_0464* | hypothetical protein | 1.12348 | 6.33E-29 | up |
| *PAS_chr4_0137* | Putative tubulin tyrosine ligase associated with P-bodies | -1.30108 | 7.25E-29 | down |
| *PAS_chr4_0138* | Small subunit of carbamoyl phosphate synthetase | -1.14985 | 1.05E-28 | down |
| *PAS_chr3_0286* | Hypothetical protein | 1.330603 | 1.60E-28 | up |
| *PAS_chr2-1_0112* | Small rho-like GTPase, essential for establishment and maintenance of cell polarity | -1.03293 | 2.26E-28 | down |
| *PAS_chr2-1_0771* | Phosphoglucomutase, catalyzes interconversion of glucose-1-phosphate and glucose-6-phospate | -1.02735 | 2.68E-28 | down |
| *PAS_chr1-1_0258* | Essential protein that associates with the contractile actomyosin ring | 1.212783 | 2.87E-28 | up |
| *PAS_chr2-1_0333* | Zeta-crystallin homolog, found in the cytoplasm and nucleus | -1.06543 | 3.19E-28 | down |
| *PAS_chr3_0986* | Serine hydrolase with sequence similarity to monoglyceride lipase (MGL) | -1.02908 | 4.48E-28 | down |
| *PAS_chr2-1_0728* | 60S ribosomal protein L28 | -1.08871 | 5.15E-28 | down |
| *PAS_chr2-1_0643* | Protein implicated in Mms22-dependent DNA repair during S phase | -1.4703 | 7.08E-28 | down |
| *PAS_chr3_1244* | hypothetical protein | -1.8112 | 1.01E-27 | down |
| *PAS_chr1-3_0310* | hypothetical protein | 1.790922 | 1.21E-27 | up |
| *PAS_chr1-1_0267* | Peptidyl-prolyl cis-trans isomerase (cyclophilin) of the endoplasmic reticulum | -1.14376 | 1.36E-27 | down |
| *PAS_chr3_0874* | Peroxisomal integral membrane peroxin | 1.512378 | 2.04E-27 | up |
| *PAS_chr3_0602* | Fatty acid elongase | -1.10244 | 2.46E-27 | down |
| *PAS_chr4_0541* | Protein that forms a complex with the Sit4p protein phosphatase and is required for its function | 1.336393 | 5.28E-27 | up |
| *PAS_chr3_0277* | 6-phosphogluconate dehydrogenase (decarboxylating) | -1.07312 | 7.72E-27 | down |
| *PAS_chr1-4_0573* | zinc finger protein | -1.03853 | 9.35E-27 | down |
| *PAS_chr1-1_0094* | Hypothetical protein | -2.18711 | 1.11E-26 | down |
| *PAS_chr2-1_0618* | Hypothetical protein | 1.187161 | 1.51E-26 | up |
| *PAS_chr2-2_0280* | Mitochondrial glycosylase/lyase | -1.28732 | 1.61E-26 | down |
| *PAS_chr2-1_0124* | Elongation factor 2 (EF-2), also encoded by EFT1 | 1.219344 | 1.63E-26 | up |
| *PAS_chr2-2_0153* | Major transcriptional repressor of DNA-damage-regulated genes | 1.543969 | 2.42E-26 | up |
| *PAS_chr2-1_0482* | 40S ribosomal protein S22 | -1.02908 | 3.32E-26 | down |
| *PAS_chr3_0821* | Protein with similarity to human cystinosin | 1.378599 | 3.72E-26 | up |
| *PAS_chr3_0712* | Basic helix-loop-helix (bHLH) protein with similarity to myc-family transcription factors | 1.02307 | 6.92E-26 | up |
| *PAS_chr4_0198* | C-8 sterol isomerase | -1.33746 | 7.32E-26 | down |
| *PAS_chr1-3_0003* | Hypothetical protein | 1.602093 | 1.25E-25 | up |
| *PAS_chr4_0315* | Histidine kinase osmosensor that regulates a MAP kinase cascade | 1.991714 | 1.29E-25 | up |
| *PAS_chr1-4_0129* | Hypothetical protein | -1.27843 | 1.46E-25 | down |
| *PAS_chr1-4_0141* | Protein with similarity to hydroxymethylpyrimidine phosphate kinases | -1.09265 | 1.52E-25 | down |
| *PAS_chr4_0584* | Aspartic protease, attached to the plasma membrane via a glycosylphosphatidylinositol (GPI) anchor | 1.038021 | 1.72E-25 | up |
| *PAS_chr1-1_0132* | Purine nucleoside phosphorylase, specifically metabolizes inosine & guanosine nucleosides | -1.10691 | 1.76E-25 | down |
| *PAS_chr3_0844* | Phosphatidylinositol transfer protein (PITP) | -1.06669 | 2.10E-25 | down |
| *PAS_chr3_0734* | Hypothetical protein | 1.582174 | 2.25E-25 | up |
| *PAS_chr3_0347* | Serine/threonine protein kinase involved in regulation of exocytosis | 1.195098 | 3.20E-25 | up |
| *PAS_chr1-4_0676* | hypothetical protein | -1.92022 | 3.42E-25 | down |
| *PAS_chr3_0623* | Ornithine carbamoyltransferase (carbamoylphosphate:L-ornithine carbamoyltransferase) | -1.23407 | 3.94E-25 | down |
| *PAS_chr3_0705* | Hypothetical protein | 1.361808 | 5.62E-25 | up |
| *PAS_chr4_0009* | Hypothetical protein | 1.031774 | 6.26E-25 | up |
| *PAS_chr1-1_0157* | Protein of unknown function, expression is sensitive to nitrogen catabolite repression | -1.09429 | 2.50E-24 | down |
| *PAS_chr3_0843* | Bifunctional enzyme | -1.14536 | 2.50E-24 | down |
| *PAS_chr1-1_0219* | 60S ribosomal protein L2 | -1.15817 | 3.49E-24 | down |
| *PAS_chr3_1065* | Putative protein of unknown function | 1.216616 | 3.67E-24 | up |
| *PAS_chr4_0647* | Low affinity vacuolar membrane localized monovalent cation/H+ antiporter | 1.240099 | 6.61E-24 | up |
| *PAS_chr2-1_0192* | Protein of unknown function | 1.02256 | 6.98E-24 | up |
| *PAS_chr1-4_0167* | Putative protein of unknown function | 1.108655 | 8.85E-24 | up |
| *PAS_chr2-2_0093* | Putative protein of unknown function | -1.30196 | 1.43E-23 | down |
| *PAS_chr4_0615* | Putative S-adenosylmethionine-dependent methyltransferase of the seven beta-strand family | -1.5743 | 1.60E-23 | down |
| *PAS_chr4_0991* | hypothetical protein | -1.44006 | 1.81E-23 | down |
| *PAS_chr1-1_0351* | Hypothetical protein | 1.141233 | 2.80E-23 | up |
| *PAS_chr3_0348* | Hypothetical protein | 1.280061 | 5.01E-23 | up |
| *PAS_chr2-1_0123* | Mitochondrial isoleucyl-tRNA synthetase, null mutant is deficient in respiratory growth | 1.236288 | 6.54E-23 | up |
| *PAS_chr3_0061* | Golgi-localized RING-finger ubiquitin ligase (E3), involved in ubiquitinating and sorting membrane p | 1.000435 | 1.01E-22 | up |
| *PAS_chr1-1_0127* | Hypothetical protein | -1.07985 | 1.07E-22 | down |
| *PAS_chr2-1_0065* | Chitin synthase III, catalyzes the transfer of N-acetylglucosamine (GlcNAc) to chitin | 1.110149 | 1.13E-22 | up |
| *PAS_chr2-1_0478* | dUTPase, catalyzes hydrolysis of dUTP to dUMP and PPi | -1.14734 | 1.24E-22 | down |
| *PAS_chr3_0209* | Cytosolic protein required for sporulation | -1.29086 | 1.39E-22 | down |
| *PAS_chr1-4_0248* | Essential protein of the mitochondrial inner membrane, component of the mitochondrial import system | -1.01095 | 1.46E-22 | down |
| *PAS_chr1-4_0276* | WD-repeat protein involved in ribosome biogenesis | -1.25124 | 1.81E-22 | down |
| *PAS_chr1-3_0299* | hypothetical protein | 1.566718 | 2.70E-22 | up |
| *PAS_chr2-1_0448* | Non-essential subunit of Sec63 complex (Sec63p, Sec62p, Sec66p and Sec72p) | -1.02165 | 2.84E-22 | down |
| *PAS_chr3_1104* | Serine-threonine kinase and endoribonuclease | 1.294294 | 2.94E-22 | up |
| *PAS_chr2-2_0291* | Essential protein of the mitochondrial intermembrane space, forms a complex with Tim9p | -1.38222 | 3.82E-22 | down |
| *PAS_chr1-1_0292* | Protein involved in rRNA processing | -1.21855 | 4.62E-22 | down |
| *PAS_chr1-1_0363* | Ubiquitin-protein ligase (E3) that interacts with Rpt4p and Rpt6p | 1.21872 | 6.37E-22 | up |
| *PAS_chr4_0711* | Hypothetical protein | 1.711782 | 7.04E-22 | up |
| *PAS_chr1-4_0442* | Putative protein of unknown function | 1.314577 | 7.80E-22 | up |
| *PAS_chr1-4_0283* | Poly(A) binding protein, part of the 3'-end RNA-processing complex | 1.853893 | 1.10E-21 | up |
| *PAS_chr1-4_0651* | Subunit of SAGA histone acetyltransferase complex | 1.620855 | 1.12E-21 | up |
| *PAS_chr4_0996* | hypothetical protein | -1.22004 | 1.28E-21 | down |
| *PAS_chr1-3_0171* | Hypothetical protein | -1.10637 | 1.40E-21 | down |
| *PAS_chr2-1_0107* | Gamma-aminobutyrate (GABA) transaminase (4-aminobutyrate aminotransferase) involved in the 4-aminobu | 1.089922 | 1.78E-21 | up |
| *PAS_chr3_0560* | Protein involved in positive regulation of both 1,3-beta-glucan synthesis and the Pkc1p-MAPK pathway | 1.157207 | 2.34E-21 | up |
| *PAS_chr2-1_0477* | Protein of unknown function, required for normal growth rate at 15 degrees C | 1.232598 | 3.43E-21 | up |
| *PAS_chr1-1_0332* | Pheromone-regulated multispanning membrane protein involved in membrane fusion during mating | -1.43946 | 3.61E-21 | down |
| *PAS_chr3_0694* | Part of actin cytoskeleton-regulatory complex Pan1p-Sla1p-End3p | 1.10341 | 4.00E-21 | up |
| *PAS_chr3_0693* | Tetrameric phosphoglycerate mutase | -1.12252 | 5.48E-21 | down |
| *PAS_chr4_0927* | hypothetical protein | 1.148737 | 5.62E-21 | up |
| *PAS_chr1-4_0669* | hypothetical protein | -1.41017 | 6.05E-21 | down |
| *PAS_chr1-4_0544* | Hypothetical protein | 1.258025 | 1.47E-20 | up |
| *PAS_chr3_1140* | Cytoplasmic light chain dynein, microtubule motor protein | -1.2984 | 2.00E-20 | down |
| *PAS_chr2-1_0458* | Peroxin 20 | 1.174705 | 2.03E-20 | up |
| *PAS_chr2-1_0241* | Component of a complex containing the Tor2p kinase and other proteins, which may have a role in regu | 1.328306 | 2.42E-20 | up |
| *PAS_chr2-1_0516* | Hypothetical protein | 1.083646 | 2.44E-20 | up |
| *PAS_chr2-1_0646* | Hypothetical protein | 1.132038 | 2.51E-20 | up |
| *PAS_chr1-4_0409* | Putative positive regulator of mannosylphosphate transferase (Mnn6p) | 1.200861 | 2.69E-20 | up |
| *PAS_chr3_0538* | Ubiquitin-protein ligase of the ER/nuclear envelope | 1.155367 | 5.93E-20 | up |
| *PAS_chr4_0468* | Transcription factor required for gene regulation in repsonse to pheromones | -1.4725 | 6.09E-20 | down |
| *PAS_chr1-4_0533* | Guanine nucleotide exchange factor (GEF or GDP-release factor) for Cdc42p | 1.063224 | 9.66E-20 | up |
| *PAS_chr3_0291* | Para hydroxybenzoate: polyprenyl transferase | -1.17067 | 9.76E-20 | down |
| *PAS_chr4_0531* | Putative protein of unknown function with similarity to F-box proteins | 1.337047 | 1.05E-19 | up |
| *PAS_chr1-4_0234* | ATP phosphoribosyltransferase (hexameric enzyme) catalyzes the first step in histidine biosynthesis | -1.11139 | 1.57E-19 | down |
| *PAS_chr1-1_0378* | Putative transmembrane protein involved in export of ammonia | -1.0366 | 2.63E-19 | down |
| *PAS_chr1-1_0385* | mitochondrial 37S ribosomal protein MRPS8 | -1.02533 | 2.65E-19 | down |
| *PAS_chr2-1_0106* | Protein necessary for structural stability of L-A double-stranded RNA-containing particles | 1.186587 | 2.92E-19 | up |
| *PAS_chr4_0892* | mitochondrial ATP synthase | -1.03447 | 3.15E-19 | down |
| *PAS_chr3_0052* | Thiamine pyrophosphokinase, phosphorylates thiamine to produce the coenzyme thiamine pyrophosphate ( | -1.04499 | 3.41E-19 | down |
| *PAS_chr2-1_0535* | Protein of the mitochondrial outer surface | 1.138623 | 3.45E-19 | up |
| *PAS_chr1-4_0207* | Hydroperoxide and superoxide-radical responsive glutathione-dependent oxidoreductase | -1.00517 | 7.47E-19 | down |
| *PAS_chr2-1_0329* | Transcriptional activator of proline utilization genes | 1.10877 | 8.40E-19 | up |
| *PAS_chr1-1_0335* | Member of the alpha/beta knot fold methyltransferase superfamily | -1.11023 | 8.55E-19 | down |
| *PAS_chr2-2_0065* | Plasma membrane multidrug transporter of the major facilitator superfamily | -1.01806 | 1.72E-18 | down |
| *PAS_chr1-4_0521* | Protein SQS1 | 1.074257 | 1.83E-18 | up |
| *PAS_chr2-2_0009* | Low-affinity Fe(II) transporter of the plasma membrane | -1.11884 | 1.88E-18 | down |
| *PAS_chr2-2_0060* | 6-phosphofructo-2-kinase, inhibited by phosphoenolpyruvate and sn-glycerol 3-phosphate, has negligib | 1.003656 | 2.02E-18 | up |
| *PAS_chr2-1_0011* | ATPase of the ATP-binding cassette (ABC) family involved in 40S and 60S ribosome biogenesis, has sim | 1.289892 | 3.39E-18 | up |
| *PAS_chr1-3_0198* | Hypothetical protein | 1.79053 | 3.48E-18 | up |
| *PAS_chr3_0115* | Transcriptional repressor and activator | 1.529562 | 6.77E-18 | up |
| *PAS_chr2-1_0562* | Protein phosphatase with specificity for serine, threonine, and tyrosine residues | 1.171527 | 6.84E-18 | up |
| *PAS_chr1-1_0235* | Protein binding phosphatidylinositol 3-phosphate | 1.389801 | 6.92E-18 | up |
| *PAS_chr1-1_0028* | Glycerol proton symporter of the plasma membrane, subject to glucose-induced inactivation | -1.25337 | 8.77E-18 | down |
| *PAS_chr3_0954* | Uroporphyrinogen decarboxylase | -1.05476 | 1.08E-17 | down |
| *PAS_chr1-1_0401* | Mitochondrial protein, putative inner membrane transporter | -1.13535 | 1.43E-17 | down |
| *PAS_chr2-2_0481* | hypothetical protein | 1.099262 | 1.50E-17 | up |
| *PAS_chr3_0484* | Putative protein of unknown function | 1.072222 | 1.56E-17 | up |
| *PAS_chr1-4_0245* | Component of the core form of RNA polymerase transcription factor TFIIH | 1.257056 | 1.91E-17 | up |
| *PAS_chr4_0298* | Component of the NuA4 histone acetyltransferase complex | 1.27308 | 2.03E-17 | up |
| *PAS_FragD_0025* | G1 cyclin, associates with Pho85p cyclin-dependent kinase (Cdk) | -1.184 | 2.11E-17 | down |
| *PAS_chr4_0060* | Evolutionarily conserved glucosamine-6-phosphate acetyltransferase | -1.00312 | 3.14E-17 | down |
| *PAS_chr1-3_0182* | Essential splicing factor | 1.680778 | 5.93E-17 | up |
| *PAS_chr3_0590* | Homeodomain-containing transcriptional repressor of PTR2 | -1.30932 | 7.39E-17 | down |
| *PAS_chr3_0801* | Cytoplasmic GTPase-activating protein for Ypt/Rab transport GTPases Ypt6p, Ypt31p and Sec4p | 1.146329 | 8.12E-17 | up |
| *PAS_chr2-1_0613* | Mitochondrial inner membrane half-type ATP-binding cassette (ABC) transporter | 1.325917 | 8.44E-17 | up |
| *PAS_chr3_0512* | Mg<sup&gt2+</sup&gt-dependent phosphatidate (PA) phosphatase | 1.066709 | 8.98E-17 | up |
| *PAS_chr1-1_0173* | Hypothetical protein | -2.30153 | 9.02E-17 | down |
| *PAS_chr3_0094* | Constituent of 66S pre-ribosomal particles, required for large (60S) ribosomal subunit biogenesis | 1.138328 | 9.30E-17 | up |
| *PAS_chr3_0413* | Protein with a potential role in pre-rRNA processing | -1.18911 | 9.37E-17 | down |
| *PAS_chr3_0024* | Plasma membrane transporter for both urea and polyamines, expression is highly sensitive to nitrogen | -1.15055 | 1.00E-16 | down |
| *PAS_chr2-1_0576* | Putative protein of unknown function | 1.229741 | 1.17E-16 | up |
| *PAS_chr3_1194* | hypothetical protein | 1.029544 | 1.23E-16 | up |
| *PAS_chr4_0075* | Hypothetical protein | -1.15451 | 1.38E-16 | down |
| *PAS_chr4_0312* | tRNA 2'-phosphotransferase | -1.09576 | 1.57E-16 | down |
| *PAS_chr1-4_0015* | GTPase | 1.196462 | 1.58E-16 | up |
| *PAS_chr2-1_0115* | Mitochondrial cytochrome-c peroxidase | -1.0593 | 2.10E-16 | down |
| *PAS_chr4_0277* | Protein that interacts with Sin3p in a two-hybrid assay | 1.17532 | 2.53E-16 | up |
| *PAS_chr1-4_0401* | transcriptional regulatory protein | 1.031531 | 2.65E-16 | up |
| *PAS_chr2-1_0048* | Ferric reductase and cupric reductase, reduces siderophore-bound iron and oxidized copper prior to u | 1.04586 | 2.93E-16 | up |
| *PAS_chr2-1_0288* | Hypothetical protein | 1.179806 | 3.29E-16 | up |
| *PAS_chr1-1_0244* | Protein of unknown function, component of the SWR1 complex | 1.858853 | 3.46E-16 | up |
| *PAS_chr4_0687* | Hypothetical protein | -1.10045 | 3.56E-16 | down |
| *PAS_chr1-4_0370* | hypothetical protein | -1.08534 | 4.25E-16 | down |
| *PAS_chr1-1_0151* | Protein that relieves transcriptional repression by binding to the Cyc8p-Tup1p corepressor | 1.152616 | 4.89E-16 | up |
| *PAS_chr1-4_0012* | TFIID subunit (145 kDa) | 1.159279 | 5.28E-16 | up |
| *PAS_chr2-1_0433* | Non-essential subunit of Sec63 complex (Sec63p, Sec62p, Sec66p and Sec72p) | -1.10848 | 6.36E-16 | down |
| *PAS_FragD_0018* | Catalytic subunit of the SWI/SNF chromatin remodeling complex involved in transcriptional regulation | 1.116388 | 7.71E-16 | up |
| *PAS_chr4_0202* | Zinc-finger transcription factor | 1.064424 | 1.03E-15 | up |
| *PAS_FragB_0027* | AP-3 complex subunit delta | 1.028748 | 1.13E-15 | up |
| *PAS_chr1-1_0364* | mRNA turnover protein MRT4 | -1.08748 | 1.38E-15 | down |
| *PAS_chr4_0745* | Subunit of TFIIH and nucleotide excision repair factor 3 complexes | 1.387228 | 1.45E-15 | up |
| *PAS_chr2-1_0389* | Sorting nexin | 1.165257 | 1.45E-15 | up |
| *PAS_chr2-1_0460* | Protein serine/threonine kinase | 1.154333 | 1.59E-15 | up |
| *PAS_chr4_0339* | Hypothetical protein | -1.33211 | 1.81E-15 | down |
| *PAS_chr1-4_0661* | hypothetical protein | 1.082652 | 2.05E-15 | up |
| *PAS_chr2-1_0798* | Putative protein of unknown function | -1.39127 | 2.51E-15 | down |
| *PAS_chr3_1195* | hypothetical protein | 1.958492 | 2.55E-15 | up |
| *PAS_chr1-1_0039* | Myb-related transcription factor | 1.01081 | 4.26E-15 | up |
| *PAS_chr3_1256* | hypothetical protein | -1.07608 | 4.70E-15 | down |
| *PAS_chr2-1_0151* | Hypothetical protein | 1.04586 | 4.82E-15 | up |
| *PAS_chr1-3_0135* | Protein that forms a complex with the Sit4p protein phosphatase | 1.19193 | 4.88E-15 | up |
| *PAS_chr3_0100* | Ornithine transporter of the mitochondrial inner membrane, exports ornithine from mitochondria as pa | -1.37085 | 5.82E-15 | down |
| *PAS_chr4_0911* | hypothetical protein | -1.29385 | 6.61E-15 | down |
| *PAS_chr2-1_0307* | Hypothetical protein | -1.07213 | 7.02E-15 | down |
| *PAS_chr4_0526* | Largest subunit of the origin recognition complex | 1.359474 | 7.44E-15 | up |
| *PAS_chr2-1_0590* | Cytoplasmic protein of unknown function predicted to encode a DNA-3-methyladenine glycosidase II | 1.260374 | 7.56E-15 | up |
| *PAS_chr4_0375* | Hypothetical protein | -1.0988 | 8.11E-15 | down |
| *PAS_chr2-1_0455* | Protein required for assembly of U2 snRNP into the spliceosome, forms a complex with Hsh49p and Hsh1 | 1.235524 | 8.60E-15 | up |
| *PAS_chr1-4_0504* | 40S ribosomal protein S29 | -1.01437 | 8.98E-15 | down |
| *PAS_chr3_1047* | Small subunit of the clathrin-associated adaptor complex AP-2 | -1.17862 | 9.54E-15 | down |
| *PAS_chr3_0963* | Hypothetical protein | 1.185606 | 9.98E-15 | up |
| *PAS_chr1-1_0355* | glycosyl hydrolase family 88, putative | -1.27226 | 1.04E-14 | down |
| *PAS_chr4_0166* | Putative transcription factor containing a C2H2 zinc finger | 1.036414 | 1.12E-14 | up |
| *PAS_chr4_0174* | Deubiquitinating enzyme anchored to the outer mitochondrial membrane | 1.632304 | 1.18E-14 | up |
| *PAS_FragD_0016* | TFIID subunit (67 kDa), involved in RNA polymerase II transcription initiation | 1.042141 | 1.23E-14 | up |
| *PAS_chr4_0527* | Non-essential putative integral membrane protein | 1.356947 | 1.47E-14 | up |
| *PAS_chr3_0063* | Putative protein of unknown function, contains a zinc finger region and has homology to human BRAP2 | 1.210506 | 1.49E-14 | up |
| *PAS_chr3_0600* | Subunit of the Set3C deacetylase complex that interacts directly with the Set3C subunit | 1.214258 | 2.27E-14 | up |
| *PAS_chr3_1246* | hypothetical protein | -1.36142 | 2.29E-14 | down |
| *PAS_FragB_0013* | Leucine-rich repeat-containing protein | 1.147875 | 2.44E-14 | up |
| *PAS_chr2-2_0315* | Putative protein of unknown function | 1.189493 | 2.68E-14 | up |
| *PAS_chr3_0420* | Hypothetical protein | 1.070799 | 3.62E-14 | up |
| *PAS_chr4_0240* | Ferric reductase, reduces siderophore-bound iron prior to uptake by transporters | -1.5112 | 4.09E-14 | down |
| *PAS_chr1-3_0147* | Protein implicated in polar growth, functionally redundant with Boi1p | 1.181669 | 4.44E-14 | up |
| *PAS_chr2-2_0277* | Protein of unknown function, localized to the mitochondrial outer membrane | -1.29987 | 5.22E-14 | down |
| *PAS_chr4_0279* | Essential tripartite DNA replication factor with single-stranded DNA-dependent ATPase | 1.115657 | 8.10E-14 | up |
| *PAS_chr1-3_0216* | Non-essential protein of unknown function | 1.190576 | 9.18E-14 | up |
| *PAS_chr2-1_0316* | Hypothetical protein | -1.33508 | 1.02E-13 | down |
| *PAS_chr1-1_0211* | Putative vacuolar protein sorting-associated protein vps13 | 1.169952 | 1.07E-13 | up |
| *PAS_chr1-1_0076* | 40S ribosomal protein S22 | -1.4434 | 1.17E-13 | down |
| *PAS_chr2-2_0103* | Serine-threonine protein kinase that is part of a glucose-sensing system | 1.083049 | 1.59E-13 | up |
| *PAS_chr2-1_0697* | Catalytic subunit of DNA polymerase zeta, which is involved in DNA repair and translesion synthesis | 1.462493 | 1.80E-13 | up |
| *PAS_chr3_0875* | Protein localized to COPII-coated vesicles, forms a complex with Erv46p | -1.02274 | 2.07E-13 | down |
| *PAS_chr2-1_0260* | Hypothetical protein | 1.003019 | 2.11E-13 | up |
| *PAS_chr3_0845* | Lyso-phosphatidylcholine acyltransferase | 1.080094 | 2.32E-13 | up |
| *PAS_chr2-2_0307* | Hypothetical protein | 1.060915 | 2.42E-13 | up |
| *PAS_chr3_0035* | Hypothetical protein | 1.414658 | 2.48E-13 | up |
| *PAS_chr2-1_0845* | hypothetical protein | 1.04384 | 3.07E-13 | up |
| *PAS_chr3_0162* | Component of a membrane-bound complex containing the Tor2p kinase and other proteins | 1.432124 | 3.18E-13 | up |
| *PAS_chr3_0138* | Hypothetical protein | -1.15307 | 3.94E-13 | down |
| *PAS_chr1-4_0560* | Hypothetical protein | -1.80453 | 4.54E-13 | down |
| *PAS_chr3_0919* | Protein of unknown function | 1.063967 | 5.12E-13 | up |
| *PAS_chr4_0971* | hypothetical protein | 1.139504 | 6.62E-13 | up |
| *PAS_chr2-1_0320* | Protein containing a UCS (UNC-45/CRO1/SHE4) domain | 1.128826 | 6.69E-13 | up |
| *PAS_chr4_0561* | Mitochondrial intermembrane space protein, forms a complex with TIm8p | -1.42539 | 6.98E-13 | down |
| *PAS_chr2-2_0113* | Inositol 1-phosphate synthase | -1.42321 | 7.24E-13 | down |
| *PAS_FragB_0019* | tRNA-dihydrouridine synthase 1 | -1.31341 | 1.23E-12 | down |
| *PAS_chr1-1_0041* | Hypothetical protein | 1.080487 | 1.30E-12 | up |
| *PAS_chr2-1_0619* | Subunit of heteropentameric Replication factor C (RF-C) | -1.07785 | 1.93E-12 | down |
| *PAS_chr2-2_0298* | Cyclin-dependent protein kinase regulatory subunit and adaptor | -1.15848 | 1.99E-12 | down |
| *PAS_chr1-4_0556* | Replication factor C | -1.03328 | 2.66E-12 | down |
| *PAS_chr2-1_0566* | Vacuolar protein of unknown function | 1.136798 | 3.44E-12 | up |
| *PAS_chr2-2_0097* | Ubl (ubiquitin-like protein)-specific protease that cleaves Smt3p protein conjugates | 1.448816 | 3.64E-12 | up |
| *PAS_chr1-4_0512* | Hypothetical protein | 1.036101 | 5.44E-12 | up |
| *PAS_chr3_0389* | Hypothetical protein | -1.52345 | 9.45E-12 | down |
| *PAS_chr1-1_0161* | N-glycosylated protein involved in the maintenance of bud site selection during bipolar budding | 1.238573 | 1.26E-11 | up |
| *PAS_chr2-1_0838* | hypothetical protein | 1.331742 | 1.31E-11 | up |
| *PAS_chr3_0518* | Predicted cytoskeleton protein | 1.025086 | 1.36E-11 | up |
| *PAS_chr1-4_0395* | Transcriptional activator of genes regulated by nitrogen catabolite repression (NCR) | 1.056863 | 1.65E-11 | up |
| *PAS_chr4_0955* | hypothetical protein | 1.209378 | 1.86E-11 | up |
| *PAS_chr1-3_0203* | Hypothetical protein | 1.121794 | 2.16E-11 | up |
| *PAS_chr1-1_0271* | Evolutionarily conserved non-essential protein present in early Golgi cisternae | -1.54045 | 2.16E-11 | down |
| *PAS_chr4_0949* | hypothetical protein | -1.15449 | 2.17E-11 | down |
| *PAS_chr2-1_0531* | Hypothetical protein | 1.209646 | 3.10E-11 | up |
| *PAS_chr3_0395* | Hypothetical protein | 1.186214 | 3.24E-11 | up |
| *PAS_chr3_1049* | Hypothetical protein | 1.140227 | 3.58E-11 | up |
| *PAS_chr1-4_0411* | Essential protein, component of a complex containing Cef1p | 1.137214 | 5.51E-11 | up |
| *PAS_chr3_1147* | hypothetical protein | 1.070299 | 7.27E-11 | up |
| *PAS_chr4_0980* | hypothetical protein | 1.371096 | 8.87E-11 | up |
| *PAS_chr1-4_0637* | Protein kti12 | -1.08516 | 1.03E-10 | down |
| *PAS_chr2-1_0447* | RING finger protein involved in proteolytic control of sumoylated substrates | 1.01429 | 1.33E-10 | up |
| *PAS_chr3_1041* | small nucleolar ribonucleoprotein SNU13 | -1.07683 | 1.64E-10 | down |
| *PAS_chr2-1_0025* | Non-essential protein of unknown function, contains ATP/GTP-binding site motif A | 1.324846 | 1.65E-10 | up |
| *PAS_chr2-1_0394* | TFIIIB B-related factor | 1.083429 | 2.27E-10 | up |
| *PAS_chr2-1_0270* | Plasma membrane protein with roles in the uptake of protoprophyrin IX and the efflux of heme | -1.38197 | 2.92E-10 | down |
| *PAS_chr1-4_0022* | Mitochondrial RNA polymerase | 1.073499 | 2.99E-10 | up |
| *PAS_chr4_0538* | Splicing factor, component of the U4/U6-U5 snRNP complex | 1.345852 | 3.20E-10 | up |
| *PAS_chr2-1_0788* | Mu3-like subunit of the clathrin associated protein complex (AP-3) | 1.266392 | 3.25E-10 | up |
| *PAS_chr2-1_0195* | Single-stranded DNA endonuclease (with Rad1p), cleaves single-stranded DNA during nucleotide excisio | 1.121609 | 3.27E-10 | up |
| *PAS_chr3_0558* | Putative protein of unknown function | -1.02158 | 3.66E-10 | down |
| *PAS_chr1-4_0035* | Hypothetical protein | 1.13779 | 5.51E-10 | up |
| *PAS_chr1-3_0111* | Hypothetical protein | -1.63259 | 8.86E-10 | down |
| *PAS_chr2-2_0309* | RNA polymerase subunit ABC14.5, common to RNA polymerases I, II, and III | -1.08529 | 9.63E-10 | down |
| *PAS_chr4_0159* | Protein with a possible role in regulating expression of nitrogen permeases | 1.124803 | 9.75E-10 | up |
| *PAS_chr1-4_0087* | Putative protein of unknown function | -1.12986 | 1.06E-09 | down |
| *PAS_chr1-1_0064* | Protein of unknown function that associates with ribosomes and has a putative RNA binding domain | -1.07281 | 1.27E-09 | down |
| *PAS_chr3_0269* | Protein required for sorting proteins to the vacuole | 1.031636 | 1.27E-09 | up |
| *PAS_chr4_0752* | Hypothetical protein | 1.053259 | 1.31E-09 | up |
| *PAS_chr3_1233* | hypothetical protein | 1.007617 | 1.71E-09 | up |
| *PAS_chr1-1_0278* | Hypothetical protein | 1.122566 | 1.83E-09 | up |
| *PAS_chr3_1079* | Hypothetical protein | 1.31259 | 2.94E-09 | up |
| *PAS_chr3_0308* | Required for a post-incision step in the repair of DNA single and double-strand breaks | 1.032844 | 2.96E-09 | up |
| *PAS_chr3_0064* | Hypothetical protein | -1.90254 | 3.81E-09 | down |
| *PAS_chr4_0854* | Hypothetical protein | -1.32826 | 4.08E-09 | down |
| *PAS_chr4_0900* | Kinesin-like protein | 1.110046 | 4.25E-09 | up |
| *PAS_chr1-1_0338* | Protein required for partitioning of the 2-micron plasmid | -1.09469 | 4.25E-09 | down |
| *PAS_chr2-2_0030* | Sorting nexin family member | 1.10169 | 4.50E-09 | up |
| *PAS_chr1-4_0481* | Hypothetical protein | 1.154712 | 5.07E-09 | up |
| *PAS_chr2-1_0446* | Hypothetical protein | -1.074 | 5.09E-09 | down |
| *PAS_chr2-1_0725* | Constituent of 66S pre-ribosomal particles, involved in 60S ribosomal subunit biogenesis | -1.26742 | 5.60E-09 | down |
| *PAS_chr3_0445* | Hypothetical protein | 1.169781 | 7.30E-09 | up |
| *PAS_chr1-4_0698* | hypothetical protein | 1.153163 | 9.02E-09 | up |
| *PAS_chr4_0833* | Protein that interacts with silencing proteins at the telomere | 1.172363 | 1.03E-08 | up |
| *PAS_chr2-2_0268* | Hypothetical protein | 1.042649 | 1.07E-08 | up |
| *PAS_chr2-1_0508* | Transcription factor involved in regulation of cell cycle progression from G1 to S phase | 1.01871 | 1.09E-08 | up |
| *PAS_chr4_0464* | AdoMet-dependent methyltransferase involved in rRNA processing and 60S ribosomal subunit maturation | 1.136968 | 1.27E-08 | up |
| *PAS_chr4_0365* | Hypothetical protein | -1.62951 | 1.30E-08 | down |
| *PAS_FragB_0058* | hypothetical protein | 1.137293 | 1.82E-08 | up |
| *PAS_chr4_0062* | Vacuolar membrane protein | 1.255557 | 1.84E-08 | up |
| *PAS_chr3_0499* | Hypothetical protein | 1.582418 | 1.89E-08 | up |
| *PAS_chr4_0919* | hypothetical protein | 1.19652 | 2.04E-08 | up |
| *PAS_chr2-2_0231* | Hypothetical protein | 1.027855 | 2.16E-08 | up |
| *PAS_chr1-4_0281* | Telobox-containing general regulatory factor | 1.131664 | 2.17E-08 | up |
| *PAS_chr3_0055* | Hypothetical protein | 1.125306 | 2.31E-08 | up |
| *PAS_FragB_0002* | Protein CSF1 | 1.025352 | 2.60E-08 | up |
| *PAS_chr1-4_0630* | Putative S-adenosylmethionine-dependent methyltransferase of the seven beta-strand family | -1.27645 | 3.46E-08 | down |
| *PAS_chr2-1_0010* | Hypothetical protein | 4.231195 | 3.75E-08 | up |
| *PAS_chr4_0160* | Kinesin-related motor protein required for mitotic spindle assembly and chromosome segregation | 1.301487 | 4.17E-08 | up |
| *PAS_chr3_0427* | Hypothetical protein | 1.136256 | 4.37E-08 | up |
| *PAS_chr2-1_0893* | hypothetical protein | -1.01156 | 4.89E-08 | down |
| *PAS_chr1-1_0207* | Hexameric DNA polymerase alpha-associated DNA helicase A involved in lagging strand DNA synthesis | 1.02552 | 5.09E-08 | up |
| *PAS_chr1-4_0453* | Hypothetical protein | 1.189359 | 5.27E-08 | up |
| *PAS_chr1-4_0251* | Putative protein of unknown function | -1.20281 | 6.61E-08 | down |
| *PAS_chr3_1253* | hypothetical protein | 1.42657 | 8.19E-08 | up |
| *PAS_chr3_0479* | Hypothetical protein | 1.34702 | 8.75E-08 | up |
| *PAS_chr3_1075* | Hypothetical protein | 1.229283 | 9.86E-08 | up |
| *PAS_chr4_0367* | Hypothetical protein | 1.121261 | 1.64E-07 | up |
| *PAS_chr2-1_0439* | DNA helicase involved in telomere formation and elongation | 1.09505 | 1.98E-07 | up |
| *PAS_FragB_0076* | hypothetical protein | -1.04305 | 2.21E-07 | down |
| *PAS_c131_0014* | Hypothetical protein | -1.1864 | 2.23E-07 | down |
| *PAS_chr2-1_0037* | Nitrilase, member of the nitrilase branch of the nitrilase superfamily | -1.25241 | 2.94E-07 | down |
| *PAS_chr1-1_0350* | Protein of the mitochondrial intermembrane space, required for acetate utilization and gluconeogenes | 1.152978 | 3.29E-07 | up |
| *PAS_chr4_1001* | hypothetical protein | 2.957768 | 3.89E-07 | up |
| *PAS_chr1-4_0606* | Membrane-associated protein | 1.004737 | 5.57E-07 | up |
| *PAS_chr2-2_0141* | Putative histone acetylase, sequence-specific activator of histone genes | 1.394454 | 6.50E-07 | up |
| *PAS_chr2-1_0879* | hypothetical protein | 1.008886 | 1.06E-06 | up |
| *PAS_chr1-4_0369* | Hypothetical protein | 1.796911 | 1.08E-06 | up |
| *PAS_chr2-1_0371* | Hypothetical protein | 1.622696 | 1.30E-06 | up |
| *PAS_chr4_0359* | Hypothetical protein | 1.326159 | 1.31E-06 | up |
| *PAS_chr1-4_0400* | hypothetical protein | 1.024045 | 1.53E-06 | up |
| *PAS_chr2-1_0534* | Hypothetical protein | 1.205094 | 1.57E-06 | up |
| *PAS_chr3_0902* | Hypothetical protein | 1.25041 | 1.67E-06 | up |
| *PAS_chr1-1_0020* | Actin-like protein | 1.235973 | 1.93E-06 | up |
| *PAS_chr2-2_0121* | Protein proposed to be involved in the modification of N-linked oligosaccharides | -1.28066 | 2.20E-06 | down |
| *PAS_chr4_0250* | DEAD-box protein required for efficient splicing of mitochondrial Group I and II introns | 1.078038 | 3.30E-06 | up |
| *PAS_chr4_0878* | Silenced copy of ALPHA2 at HML | 4.503662 | 3.31E-06 | up |
| *PAS_chr2-1_0245* | Essential protein, constituent of 66S pre-ribosomal particles | 1.115023 | 3.60E-06 | up |
| *PAS_chr3_1180* | hypothetical protein | -1.09904 | 3.85E-06 | down |
| *PAS_FragB_0023* | Vitamin H transporter 1 | -1.3704 | 4.83E-06 | down |
| *PAS_chr4_0126* | Hypothetical protein | 1.522972 | 4.94E-06 | up |
| *PAS_chr2-1_0081* | Hypothetical protein | -1.01777 | 5.32E-06 | down |
| *PAS_chr2-2_0471* | hypothetical protein | 1.048063 | 6.13E-06 | up |
| *PAS_chr4_0010* | Hypothetical protein | 1.722817 | 6.25E-06 | up |
| *PAS_chr3_0727* | Hypothetical protein | 1.430259 | 6.83E-06 | up |
| *PAS_chr2-1_0044* | Hypothetical protein | -1.00045 | 6.86E-06 | down |
| *PAS_chr3_0599* | Essential ATP-binding protein required for DNA replication, component of the pre-replicative complex | -1.00791 | 7.47E-06 | down |
| *PAS_chr3_1239* | hypothetical protein | -1.15189 | 9.11E-06 | down |
| *PAS_chr4_0388* | Mitochondrial ribosomal protein of the large subunit | -1.10339 | 1.10E-05 | down |
| *PAS_chr2-1_0586* | Guanine nucleotide exchange factor (GEF) | 1.085865 | 1.27E-05 | up |
| *PAS_chr4_0534* | Hypothetical protein | 1.076565 | 1.66E-05 | up |
| *PAS_chr1-4_0665* | hypothetical protein | -1.7651 | 1.82E-05 | down |
| *PAS_chr1-1_0090* | Putative protein of unknown function | 1.319003 | 2.00E-05 | up |
| *PAS_chr2-2_0195* | Integral ER membrane protein with type-III transmembrane domains | 1.055995 | 2.19E-05 | up |
| *PAS_chr2-2_0353* | Protein involved in G1 cell cycle arrest in response to pheromon | 1.139034 | 2.30E-05 | up |
| *PAS_chr3_0367* | Hypothetical protein | 1.514763 | 2.67E-05 | up |
| *PAS_chr3_0635* | Calpain-like cysteine protease | 1.039086 | 2.78E-05 | up |
| *PAS_chr1-3_0041* | Serine/threonine protein kinase involved in activation of meiosis | 1.407867 | 3.38E-05 | up |
| *PAS_chr2-1_0693* | Hypothetical protein | -1.11619 | 3.55E-05 | down |
| *PAS_chr4_0133* | Hypothetical protein | 1.282594 | 3.93E-05 | up |
| *PAS_chr3_1198* | hypothetical protein | 1.176242 | 4.69E-05 | up |
| *PAS_chr4_0800* | Cytoplasmic nucleoporin required for polyadenylated RNA export but not for protein import | 1.091501 | 4.72E-05 | up |
| *PAS_chr4_0693* | Hypothetical protein | 1.134677 | 5.18E-05 | up |
| *PAS_chr2-1_0475* | Hypothetical protein | 1.611634 | 5.41E-05 | up |
| *PAS_chr3_0463* | Hypothetical protein | 1.275823 | 5.64E-05 | up |
| *PAS_FragB_0018* | Hypothetical protein | -2.61828 | 5.65E-05 | down |
| *PAS_chr1-4_0377* | Hypothetical protein | 1.134623 | 7.66E-05 | up |
| *PAS_chr2-1_0108* | Hypothetical protein | 1.107732 | 8.22E-05 | up |
| *PAS_chr2-1_0620* | Hypothetical protein | -1.23417 | 8.33E-05 | down |
| *PAS_chr1-4_0374* | Subunit of an adoMet-dependent tRNA methyltransferase (MTase) complex (Trm11p-Trm112p) | -1.9628 | 8.37E-05 | down |
| *PAS_chr3_0403* | acetate--CoA ligase | -1.01748 | 8.45E-05 | down |
| *PAS_chr4_0080* | Hypothetical protein | -1.2736 | 9.16E-05 | down |
| *PAS_chr4_0382* | Mitochondrial protein of unknown function | 1.115497 | 9.32E-05 | up |
| *PAS_chr4_0371* | Hypothetical protein | -1.19027 | 0.000103 | down |
| *PAS_chr3_0550* | Meiosis-specific protein of unknown function, required for spore wall formation during sporulation | 1.029753 | 0.000108 | up |
| *PAS_chr2-2_0083* | Hypothetical protein | -1.10537 | 0.000109 | down |
| *PAS_chr2-1_0579* | Regulatory subunit of acetolactate synthase | -1.40121 | 0.000121 | down |
| *PAS_chr1-4_0373* | Hypothetical protein | 1.171689 | 0.000128 | up |
| *PAS_chr1-1_0325* | Hypothetical protein | 1.02242 | 0.000148 | up |
| *PAS_chr4_0090* | Trimethyl guanosine synthase, conserved nucleolar methyl transferase | -1.0919 | 0.000154 | down |
| *PAS_chr3_0961* | Hypothetical protein | -1.03847 | 0.000165 | down |
| *PAS_chr3_0096* | Hypothetical protein | 1.075521 | 0.000171 | up |
| *PAS_chr1-4_0105* | Involved in vesicular transport, mediates transport between an endosomal compartment and the golgi | 1.089847 | 0.000177 | up |
| *PAS_chr4_0448* | Lsm (Like Sm) protein | -1.08086 | 0.000182 | down |
| *PAS_chr2-2_0490* | hypothetical protein | 1.134875 | 0.000233 | up |
| *PAS_chr1-3_0078* | S-adenosyl-L-methionine uroporphyrinogen III transmethylase | -1.16557 | 0.000245 | down |
| *PAS_chr3_0311* | Hypothetical protein | 1.557869 | 0.000261 | up |
| *PAS_chr1-4_0344* | Essential nucleolar protein of unknown function | 1.008407 | 0.000279 | up |
| *PAS_chr3_0428* | Protein required for cytochrome c oxidase assembly | -1.1944 | 0.000326 | down |
| *PAS_chr2-1_0528* | Hypothetical protein | -1.03415 | 0.000343 | down |
| *PAS_chr3_0670* | Cytoplasmic thioredoxin isoenzyme of the thioredoxin system | 2.162071 | 0.000466 | up |
| *PAS_chr2-1_0119* | Nuclear protein that acts as a heterodimer with Aos1p to activate Smt3p (SUMO) before its conjugatio | 1.273532 | 0.000486 | up |
| *PAS_chr4_0467* | Hypothetical protein | -1.05878 | 0.000615 | down |
| *PAS_chr2-1_0554* | RNA polymerase I subunit A12.2 | -1.02189 | 0.001243 | down |
| *PAS_FragB_0057* | Exoribonuclease II, mitochondrial | 1.762574 | 0.001433 | up |
| *PAS_chr4_0747* | Constituent of small nucleolar ribonucleoprotein particles containing H/ACA-type snoRNAs | 1.977269 | 0.001489 | up |
| *PAS_chr1-3_0292* | hypothetical protein | -1.00972 | 0.001798 | down |
| *PAS_chr3_0517* | Putative GPI-anchored protein | -1.91652 | 0.003116 | down |
| *PAS_chr1-4_0696* | hypothetical protein | 1.082383 | 0.00336 | up |
| *PAS_chr1-3_0086* | Hypothetical protein | 1.314581 | 0.004066 | up |
| *PAS_chr4_0691* | Putative lipoate-protein ligase A family member | 1.438565 | 0.004244 | up |
| *PAS_chr2-1_0056* | Palmitoyltransferase that acts on the SNAREs Snc1p, Syn8p, Tlg1p and likely on all SNAREs | 1.058763 | 0.004436 | up |
| *PAS_chr4_0309* | Hypothetical protein | 1.61588 | 0.004902 | up |
| *PAS_chr4_0443* | Flippase, essential integral membrane protein that is required for translocation of Man5GlcNac2-PP-D | 1.076022 | 0.005249 | up |
| *PAS_chr2-1_0332* | Hypothetical protein | 3.591512 | 0.006002 | up |
| *PAS_chr4_0295* | Hypothetical protein | 1.059069 | 0.006783 | up |
| *PAS_chr3_0909* | Hypothetical protein | -1.0609 | 0.008817 | down |
| *PAS_chr3_0013* | Lectin-like protein with similarity to Flo1p, thought to be expressed and involved in flocculation | 1.119439 | 0.009357 | up |
| *PAS_chr3_0994* | Hypothetical protein | -1.31014 | 0.010401 | down |
| *PAS_chr1-4_0671* | hypothetical protein | 1.227437 | 0.015078 | up |
| *PAS_chr3_0103* | Hypothetical protein | -1.30558 | 0.016711 | down |
| *PAS_FragB_0043* | Hypothetical protein | 1.333639 | 0.020936 | up |
| *PAS_chr3_0708* | Essential component of the MIND kinetochore complex (Mtw1p Including Nnf1p-Nsl1p-Dsn1p) | 1.175484 | 0.021058 | up |
| *PAS_chr1-4_0111* | Hypothetical protein | -4.21574 | 0.030049 | down |
| *PAS_chr1-4_0070* | Hypothetical protein | -1.23372 | 0.033257 | down |
| *PAS_chr2-1_0704* | Protein of unknown function | 1.148677 | 0.034795 | up |
| *PAS_chr3_0886* | Hypothetical protein | -1.37463 | 0.047269 | down |
