# Supplementary material for: Economical production of Pichia pastoris single cell protein from methanol at industrial pilot scale
Source: Microb Cell Fact. 2023 Sep 28;22:198. doi: 10.1186/s12934-023-02198-9 (PMC10540378; doi:10.1186/s12934-023-02198-9)
Supplement: Supplementary file 5 — Supplementary Material 5 [file 12934_2023_2198_MOESM5_ESM.docx]

| **Reagents** | **Dosage** | **Cost**  **(CNY)** |
| --- | --- | --- |
| Strain | HTX-33-GLN1-Δ*PAS_chr4_0305* | - |
| Glycerol | 0.056 kg | 0.336 |
| Methanol | 36.25 L | 67.61 |
| KH_2_PO4 | 4.32 kg | 14.26 |
| (NH4)_2_SO4 | 2.25 kg | 3.375 |
| MgSO4•7H_2_O | 0.15 kg | 8.58 |
| Vitamin solution | 0.3 L | 50 |
| Trace metal solution | 0.6 L | 50 |
| Water | 400 L | 1.64 |
| Electricity | 700 kWh | 350 |
| pH regulators | 1 kg | 10 |
| **Total** |  | 555.8 |

**Table S5. The cost informationn of the methanol-based SCP production in the bioreactor**
