# Supplementary material for: Economical production of Pichia pastoris single cell protein from methanol at industrial pilot scale
Source: Microb Cell Fact. 2023 Sep 28;22:198. doi: 10.1186/s12934-023-02198-9 (PMC10540378; doi:10.1186/s12934-023-02198-9)
Supplement: Supplementary file 6 — Supplementary Material 6 [file 12934_2023_2198_MOESM6_ESM.docx]

**Figure S1.** The cell growth of X-33 strain in 0.5% methanol minimal medium at 28°C, 30°C, 33°C, 35°C and 37°C.
